# Feedback between motion and sensation provides nonlinear boost in run-and-tumble navigation

**DOI:** 10.1371/journal.pcbi.1005429

**Published:** 2017-03-06

**Authors:** Junjiajia Long, Steven W. Zucker, Thierry Emonet

**Affiliations:** 1 Department of Physics, Yale University, New Haven, Connecticut, United States of America; 2 Department of Molecular, Cellular and Developmental Biology, Yale University, New Haven, Connecticut, United States of America; 3 Department of Computer Science, Yale University, New Haven, Connecticut, United States of America; 4 Department of Biomedical Engineering, Yale University, New Haven, Connecticut, United States of America; Rice University, UNITED STATES

## Abstract

Many organisms navigate gradients by alternating straight motions (runs) with random reorientations (tumbles), transiently suppressing tumbles whenever attractant signal increases. This induces a functional coupling between movement and sensation, since tumbling probability is controlled by the internal state of the organism which, in turn, depends on previous signal levels. Although a negative feedback tends to maintain this internal state close to adapted levels, positive feedback can arise when motion up the gradient reduces tumbling probability, further boosting drift up the gradient. Importantly, such positive feedback can drive large fluctuations in the internal state, complicating analytical approaches. Previous studies focused on what happens when the negative feedback dominates the dynamics. By contrast, we show here that there is a large portion of physiologically-relevant parameter space where the positive feedback can dominate, even when gradients are relatively shallow. We demonstrate how large transients emerge because of non-normal dynamics (non-orthogonal eigenvectors near a stable fixed point) inherent in the positive feedback, and further identify a fundamental nonlinearity that strongly amplifies their effect. Most importantly, this amplification is asymmetric, elongating runs in favorable directions and abbreviating others. The result is a “ratchet-like” gradient climbing behavior with drift speeds that can approach half the maximum run speed of the organism. Our results thus show that the classical drawback of run-and-tumble navigation—wasteful runs in the wrong direction—can be mitigated by exploiting the non-normal dynamics implicit in the run-and-tumble strategy.

## Author summary

Countless bacteria, larvae and even larger organisms (and robots) navigate gradients by alternating periods of straight motion (runs) with random reorientation events (tumbles). Control of the tumble probability is based on previously-encountered signals. A drawback of this run-and-tumble strategy is that occasional runs in the wrong direction are wasteful. Here we show that there is an operating regime within the organism’s internal parameter space where run-and-tumble navigation can be extremely efficient. We characterize how the positive feedback between behavior and sensed signal results in a type of non-equilibrium dynamics, with the organism rapidly tumbling after moving in the wrong direction and extending motion in the right ones. For a distant source, then, the organism can find it fast.

## Introduction

Navigation up a gradient succeeds by finding those directions in which signals of interest increase. This can be difficult when the size of the navigator is small compared to the length scale of the gradient because local directional information becomes unreliable. In this case, cells [1, 2], worms [3], larvae [4], and even robots [5] often adopt a run-and-tumble strategy to navigate. During runs the organism moves approximately straight, collecting differential sensor information in one direction. Tumbles, or reorientations at zero speed, enable the organism to explore other directions. Signal levels are transduced rapidly to the motility apparatus through an internal state variable, so that increases in attractant transiently raise the probability to run longer (and tumble less) before a negative integral feedback adapts it back [6]. Classically, averaging over many runs and tumbles results in a net drift up the gradient, although this is usually rather modest because of the occasional runs in the wrong direction. We here focus on the positive feedback inherent to this strategy wherein motion up the gradient lowers the probability to tumble, which further boosts drift up the gradient. Our analysis reveals an unstudied regime in which rapid progress can be achieved. Small fluctuations in the speed of the organism along the gradient grow into large transients in the correct direction but small ones otherwise. We show that this asymmetric amplification arises from the positive feedback, which causes the eigenvectors near the adapted state of the dynamical system to become non-orthogonal, therefore leading to non-normal dynamics. The resulting large transient are further boosted by a nonlinearity that is intrinsic to the positive feedback. Such non-normal dynamics were first discovered in fluid mechanics where they were shown to play an important role in the onset of turbulence in the absence of unstable modes [7, 8].

Past theoretical studies of run-and-tumble navigation have mostly focused on what happens when adaptation dominates the dynamics (e.g. [9–13]). In this regime, the internal state of the organism exhibits small fluctuations around its mean, and mean field theory (MFT) can be applied to make predictions. This approach has been used to describe the motile behavior or populations of *E. coli* bacteria in exponential ramps [13–15] and oscillating gradients [16]. Beyond the well-understood negative feedback-dominated regime there is a large portion of physiologically relevant parameter space where the positive feedback between movement and sensation dominates the run-and-tumble dynamics. Agent-based simulations have shown that, in this case, large transient fluctuations can emerge in the internal state of an individual organism climbing a gradient, precluding the use of mean field approaches [13]. While systems of partial differential equations (PDEs) can be integrated numerically to reproduce these dynamics [17], a precise understanding of the role of the positive feedback in generating such large fluctuations and the impact of those on the performance of a biased random walk are fundamental questions that remain largely unanswered because of difficulties in obtaining analytical results.

Here we develop an analytical model of run-and-tumble gradient ascent that preserves the rich nonlinearity of the problem and incorporates the internal state, 3D-direction of motion, and position of the organism as stochastic variables. We find that large fluctuations in the internal state originate from two key mechanisms: (i) the non-normal dynamical structure of the positive feedback that enables small fluctuations to grow, and (ii) a quadratic nonlinearity in the speed along the gradient that further amplifies such transients asymmetrically. Utilizing phase space analysis and stochastic simulations, we show how these two effects combine to generate a highly effective “ratchet-like” gradient-climbing mode that strongly mitigates the classic drawback of biased random walks: wasteful runs in wrong directions. In this new regime an organism should be able to achieve drift speeds on the order of the maximum swim speed. Our results are general in that they apply to a large class of biased random walk strategies, where run speed and sampling of new directions may be modulated based on previously encountered signals.

## Results

### Minimal model of run-and-tumble navigation

Consider a random walker with an internal state variable *F* that follows linear relaxation towards the adapted state *F*_0_ over the timescale *t*_*M*_, which represents the memory duration of the random walker. We assume that the perceived signal, *ϕ*(**X**, *t*) = *ϕ*(*C*(**X**, *t*)), at position **X** and time *t* (here *C* represents the signal), is rapidly transduced to determine the value of an internal state variable *F* via a receptor with gain *N*:

$$\dot{F}=-\frac{F-F_{0}}{t_{M}}+N\left(\frac{\partial }{\partial t}+\dot{X}\cdot \nabla \right)\phi \left(X,t\right).$$ (1)

Stochastic switching between runs and tumbles depends on *F* and follows inhomogeneous Poisson statistics with probability to run *r*(*F*) = λ_*T*_/(λ_*R*_ + λ_*T*_) = 1/(1 + exp(−*HF*)), where *H* is the gain of the motor, and λ_*R*_(*F*) and λ_*T*_(*F*) are the transition rates from run to tumble and vice versa [15, 18].

During runs the speed is constant $∥\dot{X}∥=v_{0}$ and the direction of motion is subject to rotational Brownian motion with diffusion coefficient *D*_*R*_. During tumbles the speed is nil and reorientation follows rotational diffusion *D*_*T*_ > *D*_*R*_ to account for persistence effects [19]. Taken together, these two processes cause the random walker to lose its original direction at the expected rate $t_{D}^{-1}=\left(n-1\right)(rD_{R}+\left(1-rD_{T}\right)$ where *n* = 2, 3 for two- and three-dimensional motion respectively. Note that, in this minimal model we ignore possible internal signaling noise [20, 21], and all randomness comes from the rotational diffusions *D*_*R*_ and *D*_*T*_ as well as the stochastic switchings with rates λ_*R*_(*f*) and λ_*T*_(*f*). The effect of signaling noise is considered below using agent-based simulations. Since *ϕ*(*C*) can be nonlinear, Eq (1) includes possible effects of saturation of the sensory system.

Consider a static one-dimensional gradient and define the length scale of the perceived gradient as L(X)=1/||∇ϕ(X)|| and the direction of motion as $s=\hat{u}\cdot \hat{X}$. Then from Eq (1) the internal dynamics satisfies the following equations during runs and tumbles, respectively:

$$\begin{array}{cccc} & \dot{F}{|}_{run} & = & -\frac{F-F_{0}}{t_{M}}+\frac{Nv_{0}}{L(X)}s\\ & \dot{F}{|}_{tumble} & = & -\frac{F-F_{0}}{t_{M}}. \end{array}$$ (2)

We are interested in the displacement of the random walker along the gradient over timescales longer than individual runs and tumbles. In the limit where the switching timescale *t*_*S*_ = 1/(λ_*R*_ + λ_*T*_) is much shorter than the other timescales we derive from a two-state stochastic model and Eq (2) (Methods Eqs (13) and (14)):

$$\dot{r}=r\left(1-r\right)\left(\underset{\text{negative}\;\text{feedback}}{\underset{︸}{-\frac{f\left(r\right)-f_{0}}{t_{M}}}}+\underset{\text{positive}\;\text{feedback}}{\underset{︸}{\frac{r\;s}{L\left(X\right)/\left(NHv_{0}\right)}}}\right),$$ (3)

where *f* = *HF*. The first term is the negative feedback towards the adapted run probability *r*_0_ = *r*(*f*_0_). The second term shows how motion up the gradient (*s* > 0) causes the probability to run *r* to feed back on itself—when the organism is oriented up the gradient (*s* > 0), *F* increases only during runs (Eq (2)), and this increase in turn raises *r*(*F*) so that the probability that the dynamics of *F* follows $\dot{F}{|}_{run}$ rather than $\dot{F}{|}_{tumble}$ is increased, and so on. A positive feedback is thereby created with characteristic timescale *t*_*E*_ = *L*/(*NHv*_0_). Steeper gradient (smaller *L*), stronger receptor gain *N* or motor gain *H*, or faster speed *v*_0_, all lead to stronger positive feedback (shorter *t*_*E*_). This important timescale, *t*_*E*_, together with *t*_*M*_ (memory duration) and *t*_*D*_ (direction decorrelation time), effectively determines the dynamics.

Expressing time in units of *t*_*M*_, we introduce the following two non-dimensional timescales:

$$\begin{array}{cc} \tau_{E}(X) & =\frac{t_{E}(X)}{t_{M}}=\frac{L(X)}{t_{M}NHv_{0}}\\ \tau_{D}\left(f\right) & =\frac{t_{D}}{t_{M}}=\frac{1/t_{M}}{\left(n-1\right)\left(r\left(f\right)D_{R}+\left(1-r(f)\right)D_{T}\right)}. \end{array}$$ (4)

Here *τ*_*E*_ quantifies the ratio between the negative and positive feedbacks. (See Table 1 for a summary of the symbols used.) From above, we expect that the dynamics will depend on how *τ*_*E*_ and *τ*_*D*_ compare with one.

**Table 1:** Symbol definitions.

**Parameters**	
Name	Definition
*t*_*M*_	Memory, reciprocal to negative feedback
*N*	Receptor gain
*H*	Motor gain in *r*(*F*) = 1/(1 + exp(−*HF*))
*F*_0_, *f*_0_	Adapted internal state, *f*_0_ = *HF*_0_
*r*_0_	Adapted probability to run *r*(*F*_0_)
*τ*_*D*0_	*t*_*D*_(*F*_0_)/*t*_*M*_
*v*_0_	Run speed
*D*_*R*_	Rotational diffusion coefficient during runs
*D*_*T*_	Rotational diffusion coefficient during tumbles
*n*	Spatial dimension
*K*_*a*_	Dissociation constant of receptor active state
*K*_*i*_	Dissociation constant of receptor inactive state
**Independent Variables**	
Name	Definition
**X**, *X*, *x*	Position: vector, along gradient, *x* = *X*/(*v*_0_*t*_*M*_)
*t*, *τ*	Time, *τ* = *t*/*t*_*M*_
*F*, *f*	Internal state, *f* = *HF*
*s*	Swimming direction $s=\hat{u}\cdot \hat{X}=\cos\theta$
*r*	Probability to run *r*(*f*) = 1/(1 + exp(−*f*))
*v*	Normalized expected speed projected along gradient *v* = *rs*
**Dependent Variables**	
Name	Definition
*C*(**X**, *t*)	Signal concentration
*ϕ*(**X**, *t*)	Perceived signal ln((1 + *C*/*K*_*i*_)/(1 + *C*/*K*_*a*_))
*L*(**X**)	Gradient length scale 1/∥∇*ϕ*(**X**)∥
λ_*R*_(*F*)	Transition rate from run to tumble
λ_*T*_(*F*)	Transition rate from tumble to run
*t*_*S*_(*F*)	Run-tumble switching timescale 1/(λ_*R*_(*F*) + λ_*T*_(*F*))
*t_E_*(**X**)	Positive feedback timescale *L*(**X**)/(*NH*_*v*0_)
*t*_*D*_(*F*)	Direction decorrelation timescale 1/((*n* − 1)(*r*(*F*)*D*_*R*_ + (1 − *r*(*F*)*D*_*T*_))
*τ_E_*(**X**)	Ratio between negative and positive feedbacks *t_E_*(**X**)/*t_M_*
*τ*_*D*_(*F*)	Ratio between keeping direction and memory *t*_*D*_(*F*)/*t*_*M*_
*P*(*x*, *f*, *s*, *τ*)	Probability distribution of the independent variables
*p*(*f*)	Marginal probability distribution of the internal state *f*

### Exploration of the dynamical regimes

**Fig 1:**
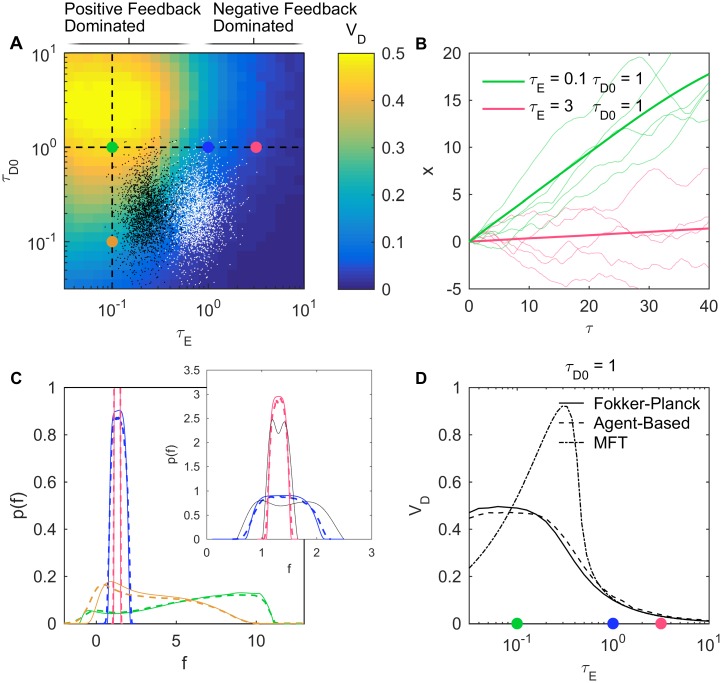
Different dynamical regimes of run-and-tumble gradient ascent. (A) Drift speed *V*_*D*_ of simulated *E. coli* cells swimming in static exponential gradients as a function of *τ*_*E*_ and *τ*_*D*0_. Green, blue, and red: *τ*_*D*0_ = 1 and *τ*_*E*_ = 0.1, 1, 3, respectively. Orange: *τ*_*E*_ = 0.1 and *τ*_*D*0_ = 0.1 (dashed line: guides to the eye). White/black: sampling of a wild type population [22] near the bottom/top of a linear gradient. (B) Classical (red) vs. rapid climbing (green) trajectories. *x* = *X*/(*v*_0_*t*_*M*_) vs. time *τ* = *t*/*t*_*M*_ for cells in the positive-feedback- (green) and negative-feedback-dominated (red) regime (thin: 5 samples; thick: mean over 10^4^ samples). (C) Marginal probability distribution of the internal variable at steady state $\bar{p}\left(f\right)$; solid: numerical solution of Eq (5); dashed: sampled distribution from agent-based simulation; colors: same parameter values as in A. Inset: zoomed view with second order analytical approximations (Methods Eq (35)) in black. *r*_0_ = 0.8 and *D*_*T*_/*D*_*R*_ = 37 in all simulations. (D) Comparison of different methods to calculate *V*_*D*_ as a function of *τ*_*E*_ keeping *τ*_*D*0_ = 1 fixed. Solid: numerical integration of Eqs (5) and (6); dashed: agent-based model simulations; dash-dot: MFT (Methods Eq (43)). Details in Methods.

To explore how run-and-tumble dynamics depend on *τ*_*E*_ and *τ*_*D*_, we used a previously published stochastic agent-based simulator of the bacteria *E. coli* that reproduces well available experimental data on the wild-type laboratory strain RP437 ([15, 22] and S1 Appendix). In this case the internal state *F* represents the free energy of the chemoreceptors. Since *E. coli* approximately detects log-concentrations (S1 Appendix Eq (S11)), we simulated an exponential gradient so that *τ*_*E*_ is a constant. In this case the cells reach steady state with a constant drift speed *V*_*D*_. Calculating *V*_*D*_ from 10^4^ simulated trajectories for a range of *τ*_*E*_ and *τ*_*D*0_ = *τ*_*D*_(*r*_0_) values reveals that cells climb the gradient much faster when the positive feedback dominates (*τ*_*E*_ < 1) (Fig 1A). The trajectories of individual cells resembled that of a ratchet that moves almost only in one direction (Fig 1B green). In contrast, when the negative feedback dominates (*τ*_*E*_ > 1) the trajectories exhibit both up and down runs of similar although slightly biased lengths (Fig 1B red). *V*_*D*_ also depends on *τ*_*D*_ and peaks when the direction decorrelation time is on the same order as the memory duration (*τ*_*D*_ ≃ 1), consistent with previous studies [11, 23, 24]. In these simulations the adapted probability to run *r*_0_ = 0.8 and the ratio *D*_*T*_/*D*_*R*_ = 37 were kept constant. Changing these values did not change the main results (S1A–S1C Fig).

In a wild type population, individual isogenic cells will have different values of *τ*_*E*_ and *τ*_*D*0_ due to cell-to-cell variabilities in swimming speed and in the abundance of chemotaxis proteins [22, 25, 26]. In a recent experimental study, the phenotype and performance of individual wild type cells (RP437 strain) was quantified by tracking cells swimming up a static quasi-linear gradient of methyl-aspartate (varying from 0 to 1 *mM* over 10 *mm*). This experiment revealed large differences among the performances of individual cells within the isogenic population [22], which could be reproduced by complementing the model of bacterial chemotaxis just described with a simple model of noisy gene expression (Fig 2 in [22]). To examine in which region of the (*τ*_*E*_, *τ*_*D*0_) space these cells might have been operating, we used this same model (complemented with diversity in rotational diffusion coefficients *D*_*R*_ and *D*_*T*_ due to variations in cell length; see S1 Appendix) with the same parameter values to run simulations of 16,000 cells climbing the experimentally measured (Fig 1A). We find that even in this relatively shallow gradient some cells might have been operating in the positive-feedback-dominated regime, especially near the bottom of the gradient (black dots). As the cells climb the gradient, *τ*_*E*_ becomes larger (white dots) because, as concentration increases, the log-sensing cells in the quasi-linear gradient face a shallower gradient, and thus weaker positive feedback.

### Positive feedback between motion and sensation generates large internal state fluctuations and fast drift

To better understand the origin of the fast drift speed and its associated “ratchet-like” behavior, we examine the relationship between the drift speed *V*_*D*_ and the statistics of the internal state *f*. Using *t*_*M*_ as the unit of time and *v*_0_*t*_*M*_ as the unit of length we derive a Fokker-Planck equation for the probability *P*(*x*, *f*, *s*, *τ*) that at time *τ* = *t*/*t*_*M*_ the cell is at position *x* = *X*/(*v*_0_*t*_*M*_) with internal state *f* and orientation *s* (Methods Eq (14)):

$$\partial_{\tau }P=-\partial_{f}\left(\left(-\left(f-f_{0}\right)+\frac{r(f)s}{\tau_{E}\left(x\right)}\right)P\right)+\frac{{\hat{L}}_{s}P}{\left(n-1\right)\tau_{D}\left(f\right)}-\partial_{x}\left(r(f)sP\right).$$ (5)

Here ${\hat{L}}_{s}={\left(1-s^{2}\right)}^{\frac{3-n}{2}}\partial_{s}\left({\left(1-s^{2}\right)}^{\frac{n-1}{2}}\partial_{s}\right)$ is the rotational diffusion operator on the (*n* − 1)-sphere. All symbols used are summarized in Table 1.

For simplicity we consider a log-sensing organism swimming in a static exponential gradient. In this case, *τ*_*E*_(*x*) = *τ*_*E*_ is constant (more complex gradient profiles and the effect of receptor saturation are considered later in the paper). Therefore the positive feedback becomes independent of position and the system can reach a steady state drift speed. Separating the variable *x* and integrating over *x* we obtain (Methods Eq (17))

$$\begin{array}{c} V_{D}=\tau_{E}\bar{\left\langle f-f_{0}\right\rangle }, \end{array}$$ (6)

where 〈⋅〉 represents averaging over *f* and *s* and the bar indicates steady state. Eq (6) indicates that the drift speed is determined by the steady state marginal distribution $\bar{p}\left(f\right)$. To find an analytical expression for $\bar{p}\left(f\right)$, we expand the steady state joint distribution $\bar{P}\left(f,s\right)$ in orthonormal eigenfunctions of the angular operator ${\hat{L}}_{s}$—the first two coefficients are the marginal distribution $\bar{p}\left(f\right)$ and the first angular moment ${\bar{p}}_{1}\left(f\right)/\sqrt{n}=\int \bar{P}sds$—and discard higher orders to obtain a closed system of equations. The analytical solution for the steady state marginal distribution $\bar{p}\left(f\right)$ reads

$$\bar{p}\left(f\right)=\frac{1}{W}\frac{\frac{r(f)}{\tau_{E}}}{\frac{1}{n}{\left(\frac{r(f)}{\tau_{E}}\right)}^{2}-{\left(f-f_{0}\right)}^{2}}\exp\left(-\int^{f}\frac{\frac{f_{1}-f_{0}}{\tau_{D}\left(f_{1}\right)}}{\frac{1}{n}{\left(\frac{r(f_{1})}{\tau_{E}}\right)}^{2}-{\left(f_{1}-f_{0}\right)}^{2}}df_{1}\right),$$ (7)

where *W* is a normalization constant. The full derivation is provided in Methods Eqs (25)–(27), together with an interpretation of the distribution as a potential solution $\bar{p}\left(f\right)\propto \exp(-V\left(f)\right)$ where *V*(*f*) is the “potential”. We also examine how the shape of the potential depends on *τ*_*E*_ and *τ*_*D*_.

The solution $\bar{p}\left(f\right)$ is plotted in Fig 1C. When the negative feedback dominates (*τ_E_* ≳ 1) the distribution is sharply peaked and nearly Gaussian with variance $\sigma^{2}=\tau_{D0}r_{0}^{2}/n\tau_{E}^{2}$ (Methods Eq (32)) and its mean barely deviates from the adapted state *f*_0_ (Fig 1C red and blue). Substituting $\bar{p}\left(f\right)$ into Eq (6) and taking the limit *τ*_*E*_ ≫ 1 yields known MFT results [11–13] (Methods Eq (43)). When the positive feedback dominates (*τ*_*E*_ ≪ 1) the distribution $\bar{p}\left(f\right)$ now exhibits large asymmetrical deviations (Fig 1C green) between the lower and upper bounds *f*_*L*_ and *f*_*U*_, which satisfy the relations *f*_*L*_ = *f*_0_ − *r*(*f*_*L*_)/*τ*_*E*_ and *f*_*U*_ = *f*_0_ + *r*(*f*_*U*_)/*τ*_*E*_. For small *τ*_*E*_ the lower bound decreases as *f*_*L*_ → ln *τ*_*E*_ whereas the upper bound increases as *f*_*U*_ → 1/*τ*_*E*_ (Methods Eq (46)). MFT becomes inadequate in this regime, as recently suggested by 1D approximations [17]. When the positive feedback dominates, matching the memory of the cell with the direction decorrelation time becomes important: keeping the direction of motion long enough (*τ_D_* ≳ 1) allows the distribution to develop a peak near *f*_*U*_ (Fig 1C green), which according to Eq (6) results in higher drift speed (S2 Fig). We verified the approximate analytical solution $\bar{p}\left(f\right)$ captures the run-and-tumble dynamics well by plotting it against the distribution of *f* obtained from the agent-based simulations (Fig 1C). Integrating $\bar{p}\left(f\right)$ according to Eq (6) predicts well the drift speed for all *τ*_*E*_ (Fig 1D), including where the positive feedback dominates (*τ*_*E*_ < 1).

### Nonlinear amplification of non-normal dynamics generates long runs uphill but short ones otherwise

**Fig 2:**
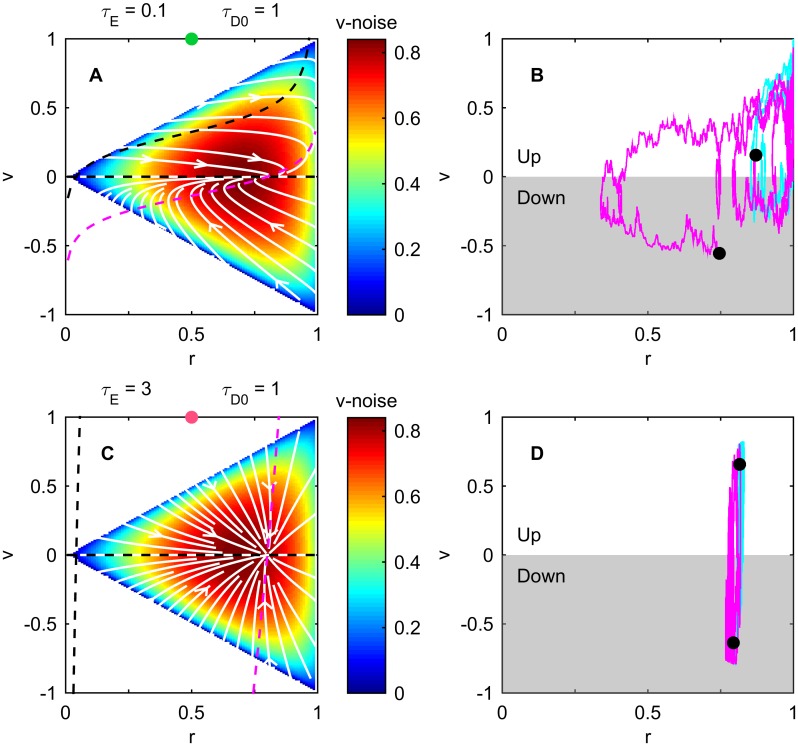
Non-normal dynamics enables large asymmetric transients in internal state. (A) Phase space diagram of Eq (8) when the positive feedback dominates, *τ*_*E*_ = 0.1. White: streamlines without noise; magenta: the *r*-nullcline where *dr*/*dτ* = 0; black: the two *v*-nullclines where *dv*/*dτ* = 0. Heat map: noise magnitude of *dv*/*dτ* ($\sqrt{\left(r^{2}-v^{2}\right)/\tau_{D}\left(r\right)}$ in Eq (8)). (B) Two example trajectories starting in positive (cyan) or negative (magenta) direction. Each trajectory starts from black and lasts over the same time period of *τ* = 10. See also S1 Movie. (C,D) Same as A,B except in the negative-feedback-dominated regime, *τ*_*E*_ = 3. When the positive feedback dominates (*τ*_*E*_ = 0.1, A), the streamlines (white) are highly asymmetric around the fixed point. They tend to bring the system transiently towards *r* = 1 and *v* = 1—a result of both non-normal dynamics (non-orthogonal eigenvectors near the fixed point) and nonlinear positive feedback (growth towards *v* = 1 away from the fixed point)—before eventually falling back to the fixed point. High noise near the fixed point causes the system to quickly move away from it (magenta in B). Low noise in the upper right corner (*r* = 1 and *v* = 1) facilitates longer runs in the correct direction (cyan in B). Taken together, these effects result in a fast “ratchet-like” gradient climbing behavior. In contrast, when the negative feedback dominates (*τ*_*E*_ = 0.1, C) the streamlines all point back directly to the fixed point and small deviations do not grow (cyan and magenta in D). Details in Methods.

In the fast gradient climbing regime (*τ*_*E*_ ≪ 1) trajectories resemble that of a ratchet (Fig 1B). To gain mechanistic insight into this striking efficiency we examined the Langevin system equivalent to the Fokker-Planck Eq (5). Defining *v* = *rs* as the normalized run speed projected along the gradient, we change variables from (*f*, *s*) to (*r*, *v*) and obtain (Methods Eqs (47)–(52)):

$$\begin{array}{cc} \frac{dr}{d\tau }= & \;r\left(1-r\right)\left(-\left(f\left(r\right)-f_{0}\right)+\frac{v}{\tau_{E}}\right)\\ \frac{dv}{d\tau }= & \;v\left(1-r\right)\left(-\left(f\left(r\right)-f_{0}\right)+\frac{v}{\tau_{E}}\right)-\frac{v}{\tau_{D}\left(r\right)}+\sqrt{\frac{\left(r^{2}-v^{2}\right)}{\tau_{D}\left(r\right)}}\eta \left(\tau \right), \end{array}$$ (8)

where *v* = *dx*/*dτ* and *η*(*τ*) denotes delta-correlated Gaussian white noise. The nullclines of the system (Fig 2A and 2C) intersect at the only stable fixed point (*r*, *v*) = (*r*_0_, 0) of Eq (8) where the eigenvalues of the relaxation matrix

$$\left[\begin{array}{cc} -1 & \left(1-r_{0}\right)r_{0}/\tau_{E}\\ 0 & -1/\tau_{D0} \end{array}\right]$$ (9)

are both negative (Methods Eq (53)). Stochastic fluctuations due to rotational diffusions *D*_*R*_ and *D*_*T*_ (heat maps in Fig 2A and 2C) continuously push the system away from the fixed point. The magnitude of these fluctuations is large near the fixed point, causing the system to quickly move away. Fluctuations are smaller near *r* = 1 and *v* = 1, enabling the organism to climb the gradient at high speed for a longer time. Net drift results from spending more time in the region where *v* > 0.

Stochastic excursions in the (*r*, *v*)-plane away from the fixed point exhibit distinctive trajectories depending on the value of *τ*_*E*_. When the positive feedback dominates (*τ*_*E*_ ≪ 1; Fig 2A) the eigenvectors of the relaxation matrix, (1, 0)^*T*^ and ${\left(\frac{(1-r_{0})r_{0}}{\tau_{E}}\frac{\tau_{D0}}{\tau_{D0}-1},1\right)}^{T}$, are highly non-orthogonal. This defines a non-normal dynamics that enables linear deviations to grow transiently [7, 8] to feed the nonlinear positive feedback (*v*^2^ term second line in Eq (8)) leading to large deviations. Importantly, this only happens for runs that start in the correct direction. If the run is in the wrong direction the linear deviation does not grow (Fig 2B; see also S1 Movie). Asymmetry arises because the *v*^2^ term is always positive. Similar selective amplification properties are observed in neuronal networks, where non-normal dynamics enables the network to respond to certain signals while ignoring others (including noise) [27, 28]. Thus, a random walker running in the correct direction is aided by the positive feedback, which pushes its internal dynamics towards the upper right corner of the phase plane where *r* = 1 and *v* = 1. If, instead, the run is in the wrong direction (*v* < 0), the nonlinearity pushes the system back into the high noise region near the fixed point where it will rapidly pick a new direction (Fig 2B).

In contrast, when the negative feedback dominates (*τ*_*E*_ ≳ 1; Fig 2C), the eigenvectors become nearly orthogonal. Linear deviations from the fixed point simply relax to the fixed point regardless of the initial direction of the run. Thus runs up and down the gradient are only marginally different in length, resulting in a small net drift (Fig 2D). This key difference between the positive and negative feedback regimes is reflected in the flow field (white curves in Fig 2A and 2C).

### Receptor saturation, varying gradient length scales, and trade-offs

**Fig 3:**
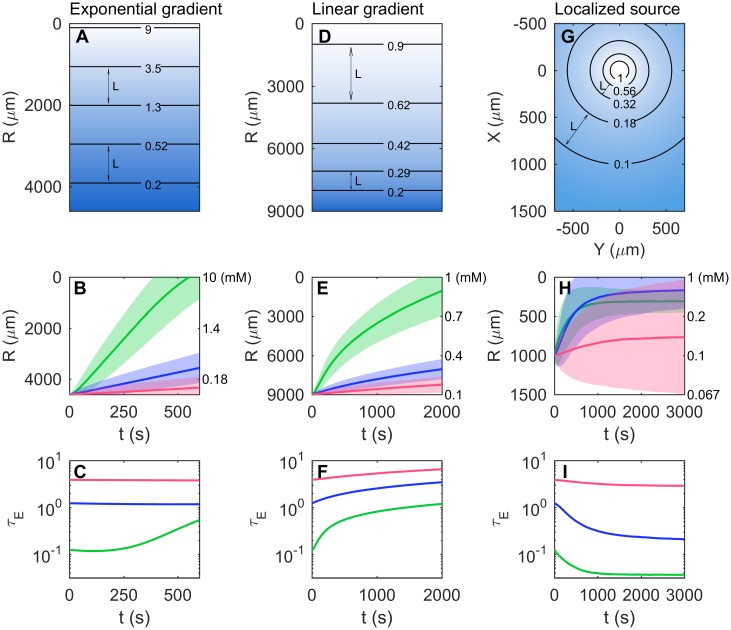
Environmental context, length scales, and receptor saturation. (A-C) Exponential gradient. (A) Schematic of a gradient of methyl-aspartate *C* = *C*_0_ exp(−*R*/*L*_0_) with length scale *L*_0_ = 1000 *μm* and source concentration *C*_0_ = 10 *mM*. Contour lines show logarithmically spaced concentration levels in units of *mM*. Contour spacing illustrates constant *L* = 1/|∂_*R*_ ln *C*| = *L*_0_. (B) The mean trajectory over 10^4^ *E. coli* cells of the position *R* (in real units *μm*) as a function of time *t* (in *s*) when receptor saturation is taken into account. Initial values of *τ*_*E*_ are 0.1 (green), 1 (blue) or 3 (red). The shadings indicate standard deviations. The labels on the right axis show the concentration in *mM* at each position. (C) Corresponding time trajectories of the values of *τ*_*E*_ at mean positions. (D-F) Linear gradient. Similar to A-C but for *C* = *C*_1_ − *a*_1_*R* where the source concentration is *C*_1_ = 1 *mM* and decreases linearly at rate *a*_1_ = 0.0001 *mM*/*μm* with distance *R* from the source. Contour spacing decreases with distance from the source (at the top), illustrating decreasing *L* = 1/|∂_*R*_ ln *C*| = *C*/*a*_1_ = *C*_1_/*a*_1_ − *R*. (G-I) Localized source. Similar to A-C but for a constant source concentration (*C*_2_ = 1 *mM*) within a ball of radius *R*_0_ = 100 *μm* and for *R* > *R*_0_, the concentration is *C* = *C*_2_*R*_0_/*R* (the steady state solution to the standard diffusion equation ∂_*t*_*C* = ∇^2^*C* without decay), decreasing with radial distance as 1/*R* away from the source. Contour spacing increases away from the source (at the origin), illustrating increasing *L* = 1/|∂_*R*_ ln *C*| = *R*.

For simplicity in our analytical derivations we assumed the environment was a constant exponential gradient with concentrations in the (log-sensing) sensitivity range of the organism. Here we explore what happens when the organism encounters concentrations beyond its sensitivity range. For wild type *E. coli* the change in the free energy of the chemorecetor cluster due to ligand binding is proportional to ln((1 + *C*/*K*_*i*_)/(1 + *C*/*K*_*a*_)) (S1 Appendix Eq (S8)). Therefore the receptor is log-sensing to methyl-aspartate only for concentrations between *K*_*i*_ ≪ *C* ≪ *K*_*a*_, where *K*_*i*_ = 0.0182 *mM* and *K*_*a*_ = 3 *mM* are the dissociation constants of the inactive and active states of the receptor. When *C* < *K*_*i*_ the receptor senses linear concentration [29], whereas when *C* > *K*_*a*_ the receptors saturate [30–33]: as a cell approaches a high concentration source its sensitivity decreases (S1 Appendix Eq (S10)). This in turn increases the value of *τ*_*E*_. Simulations in an exponential gradient show that this effect results in an eventual slow-down as the cell approaches the source (Fig 3A–3C).

Realistic gradients are typically limited in spatial extent and often are not exponential, in which case *L* and therefore *τ*_*E*_ are different in different regions. *L* is long near the source in a linear gradient, for example, and decreases linearly with distance from the source. Simulations show that the cell initially climbs the gradient fast but later slows down as the gradient length scale *L* increases and *τ*_*E*_ increases (Fig 3D–3F). On the contrary, for a static localized source in three dimensions, *L* is short near the source but increases linearly with distance from it (Methods). Thus, *τ*_*E*_ decreases and the cell accelerates as it approaches the source (Fig 3G–3I). Comparing cells in various dynamical regimes (different values of *τ*_*E*_) across these different gradients suggests that a lower value of *τ*_*E*_ results in faster gradient ascent.

When entering a food gradient, it is natural to try to climb as fast as possible. However this strategy could create a problem: the longer runs implied by the positive feedback mechanism could propel the accelerating *E. coli* beyond the nutrient source. This is the case in Fig 3E, where cells with the lowest *τ*_*E*_ (green) reach the source first but overshoot slightly; they settle, on average, at a further distance than those with intermediate *τ*_*E*_ (blue). Thus there is a trade-off between transient gradient climbing and long-term aggregating, as previously observed [13, 15, 23]. In nature, as chemotactic bacteria live in swarms, chasing and eating nutrient patches driven by flows and diffusions while new plumes of nutrients are constantly created by other organisms [2, 34], the actual environments experienced by bacteria are far more complex. The trade-off we found here hints that in these random small fluctuating gradients [11, 16, 35–37] the bacteria should not aim for maximal drift speed but need to deal with this trade-off to avoid overshoot. In general, natural environments will be complex, with a variety of different sources and gradients, implying different parameter domains will be optimal for *E. coli* at different times. Such phenotypic diversity may well confer an advantage [15, 37–41].

## Discussion

Our results illustrate the surprisingly new capabilities that can emerge when living systems exploit the full nonlinearity inherent within an otherwise simple and widely used strategy. For the particular case of bacterial chemotaxis we showed that cells that swim fast, have long memory (adaptation time), or large signal amplification, are likely to exhibit “ratchet-like” climbing behavior in a positive-feedback-dominated regime, even in shallow gradients. As we showed from simulations using a model that fits experimental data, this regime should be accessible to wild-type bacterial populations. Actually identifying these “ratcheting” cells from experimental trajectories would require observing them for a sufficient time (*T* ≫ *t*_*M*_, *t*_*D*_, *t*_*E*_) and in a sufficiently steep gradient over the distance traveled (Δ*X* = *V*_*D*_*T* ∼ 0.5*v*_0_*T*). Using parameter estimates from [13, 20], for *t*_*E*_ < *t*_*D*_ ≃ *t*_*M*_ ∼ 10 *s* we take *T* ∼ 200 *s*, and for *v*_0_ = 20 *μm*/*s* we get Δ*X* = 2 *mm*. To see how this compares with existing experimental setup with a quasi-linear gradient varying from 0 to 1 *mM* over 10 *mm* [22], we note that the black dots in Fig 1A show that some cells located 1.5 *mm* away from 0 concentration can operate in the positive-feedback-dominated regime. Thus, using the same setup as in [22] these requirements would be satisfied near the bottom of the gradient if the source concentration was increased to 3 *mM*.

It is common to make simplifying assumptions to facilitate analysis, but we do not believe that ours are limiting. We showed with simulations that our results hold (S1 Fig for details) when we take into account: (i) different values of *r*_0_ and *D*_*T*_/*D*_*R*_; (ii) the limited range of the receptor sensitivity [15, 18] (S1 Appendix Eq (S10)); (iii) possible nonlinearities (S1 Appendix Eq (S4)) and asymmetries of adaptation rates [14, 42]. A hallmark of *E. coli* chemotaxis is that, in the absence of a gradient, run-and-tumble behavior adapts back to prestimulus statistics [6, 43]. These robust properties of integral feedback control [6] remain in place in our study because the transients originate from non-normal dynamics around the stable fixed point. The boost from positive feedback described here is independent from other mechanisms that can enhance drift up a gradient such as imperfect adaptation in the response to some amino acids [44] and stochastic fluctuations in the adaptation mechanism [20, 21]. The latter has been shown to enhance chemotactic performance in shallow gradients by transiently pushing the system into a regime of slower direction changing provided it is running up the gradient. There are some similarities between the effect of signaling noise and the positive feedback mechanism presented here: both can affect drift speed by causing long-lasting asymmetries in the internal state when running up the gradient. In S3 Fig we show using simulations that signaling noise in the adaptation mechanism does not change our conclusion that the drift speed is maximal in the positive-feedback-dominated regime. Depending on the region of the (*τ*_*E*_, *τ*_*D*_) parameter space, the signaling noise can either enhance the drift speed by less than 10% or reduce it by up to 30%.

The fact that non-normal dynamics might be exploited to boost runs in the correct direction parallels recent findings in neuroscience [27] that suggest neuronal networks use similar strategies to selectively amplify neural activity patterns of interest. Thus, non-normal dynamics could be a feature that is selected for in living dynamical systems. Although we used bacterial chemotaxis as an example, our results do not depend on the specific form of the functions *r*(*f*) and *t*_*D*_(*f*), provided they are increasing. Therefore our findings should be applicable to a large class of biased random walk strategies exhibited by organisms when local directional information is unreliable. In essence, any stochastic navigation strategy requires a memory, *t*_*M*_, to make temporal comparisons, a reorientation mechanism, *t*_*D*_, to sample new directions, and external information, *t*_*E*_, relayed to decision-making circuitry through motion and signal amplification. Our theoretical contribution showed the (surprisingly) diverse behavioral repertoire that is possible by having these work in concert. In retrospect, perhaps this should not be surprising given the diverse environments in which running-and-tumbling organisms can thrive.

## Methods

### Agent-based simulation

#### Chemotaxis pathway model

A detailed description of the chemotaxis model and agent-based simulations is provided in S1 Appendix (parameters in S1 Table). Briefly, the agent-based simulations were performed using Euler’s method to integrate a standard model of *E. coli* chemotaxis [12–14, 18, 20] in which the cell relays information from the external environment to the flagellar motor through a signaling cascade triggered by ligand-binding receptors. The receptors are described by a two-state model where the activity *a* is determined by the free energy difference *F* between the active and inactive states, which is determined by both the ligand concentration *C* and the receptors’ methylation level *m*. At each time step, the cell moves forward or stays in place according to its motility state (run or tumble), which also determines whether its direction changes with rotational diffusion coefficients *D*_*R*_ or *D*_*T*_. At the new position changes in ligand concentration *C* cause changes in free energy *F* and thus activity *a*, and the methylation state adapts to compensate that change to maintain a constant activity. The updated value of the free energy *F* then determines the switching rates between the clockwise and counter-clockwise rotation of the flagellar motor state, which in turn determines the motility state of the cell according to rules and parameters in [20], completing one time step.

#### Noisy gene expression model

In Fig 1A we considered a wild type population in the scatter plot. To generate a population with realistic parameters, we used a recent model [22] of phenotypic diversity in *E. coli* chemotaxis that reproduces available experimental data on the laboratory strain RP437 climbing a linear gradient of methyl-aspartate. In this model individual cells have different abundances of the chemotaxis proteins (CheRBYZAW) and receptors (Tar, Tsr). These molecular abundances then determine the memory time *t*_*M*_ and the adapted probability to run *r*_0_ [15]. The run speed was different among cells and sampled from a Gaussian distribution to match experimental observations [22]. Rotational diffusion coefficients were also distributed to reflect differences in cell length.

### Derivation of Eqs (3)–(5), the Fokker-Planck equation model in the fast switching limit

We define $P_{R}\left(X,\hat{u},F,t\right)$ and $P_{T}\left(X,\hat{u},F,t\right)$ as the probability distributions at time *t* to be running or tumbling at position **X** in direction $\hat{u}$ with internal variable *F*. As described, there is Poisson switching between runs and tumbles with rates λ_*R*_(*F*) and λ_*T*_(*F*), runs and tumbles follow rotational diffusion with *D*_*R*_ and *D*_*T*_, and motion is constant in runs and 0 in tumbles. Thus we construct a two-state stochastic master equation model [45]

$$\begin{array}{cc} \partial_{t}P_{R}= & -\partial_{F}\left(\dot{F}{|}_{run}P_{R}\right)-\nabla \cdot \left(v_{0}\hat{u}P_{R}\right)+\nabla_{\hat{u}}^{2}\left(D_{R}P_{R}\right)-\lambda_{R}P_{R}+\lambda_{T}P_{T}\\ \partial_{t}P_{T}= & -\partial_{F}\left(\dot{F}{|}_{tumble}P_{T}\right)+\nabla_{\hat{u}}^{2}\left(D_{T}P_{T}\right)+\lambda_{R}P_{R}-\lambda_{T}P_{T}, \end{array}$$ (10)

where $\dot{F}{|}_{run,tumble}$ are defined in Eq (2).

Since the gradient varies in one direction only we focus on motion in the gradient direction and integrate the probability over all other directions. Thus $\nabla \cdot \hat{u}=s\partial_{X}$ and $\nabla_{\hat{u}}^{2}={\hat{L}}_{s}$, the polar angle part of the rotational diffusion operator on the (*n* − 1)-sphere. To derive the analytical form of ${\hat{L}}_{s}$ we note in *n*-dimensional space we can iteratively write down the Laplace-Beltrami operator [46] as

$$\begin{array}{c} \nabla_{S^{n-1}}^{2}={\left(\sin\theta \right)}^{2-n}\partial_{\theta }\left({\left(\sin\theta \right)}^{n-2}\partial_{\theta }\right)+{\left(\sin\theta \right)}^{-2}\nabla_{S^{n-2}}^{2}, \end{array}$$ (11)

where 0 < *θ* < *π* is the polar angle. In a one-dimensional gradient we define the gradient direction as the polar axis, thus $s=\hat{u}\cdot \hat{X}=\cos\theta$. We can write $\sin\theta =\sqrt{1-s^{2}}$ and $\partial_{\theta }=-\sqrt{1-s^{2}}\partial_{s}$. Then the polar angle part is

$$\begin{array}{c} L_{s}={\left(1-s^{2}\right)}^{\frac{3-n}{2}}\hat{\partial_{s}}\left({\left(1-s^{2}\right)}^{\frac{n-1}{2}}\partial_{s}\right). \end{array}$$ (12)

Using the definitions of the normalized internal state *f* = *HF*, of the timescale of switching between runs and tumbles *t*_*S*_ = 1/(λ_*R*_ + λ_*T*_) [45], and of the probability to run *r* = λ_*T*_/(λ_*R*_ + λ_*T*_), we obtain

$$\begin{array}{cc} \partial_{t}P_{R}= & -\partial_{f}\left(\left(-\frac{f-f_{0}}{t_{M}}+\frac{NHv_{0}}{L}s\right)P_{R}\right)+D_{R}{\hat{L}}_{s}P_{R}\\ & -\frac{1-r}{t_{S}}P_{R}+\frac{r}{t_{S}}P_{T}-s\partial_{X}\left(v_{0}P_{R}\right)\\ \partial_{t}P_{T}= & -\partial_{f}\left(-\frac{f-f_{0}}{t_{M}}P_{T}\right)+D_{T}{\hat{L}}_{s}P_{T}\\ & +\frac{1-r}{t_{S}}P_{R}-\frac{r}{t_{S}}P_{T}. \end{array}$$ (13)

If we assume the switching terms with $t_{S}^{-1}$ in Eq (13) dominate, the probabilities to be running and tumbling equilibrate on a much faster timescale than the other ones. Therefore we can let *P* = *P*_*R*_ + *P*_*T*_ and can approximate the actual probability to run as *P*_*R*_/*P* ≈ *r*. Adding the two equations above yields the Fokker-Planck equation:

$$\begin{array}{cc} \partial_{t}P\approx & -\partial_{f}\left(\left(-\frac{f-f_{0}}{t_{M}}+\frac{rs}{L/NHv_{0}}\right)P\right)\\ & +\left(rD_{R}+\left(1-r\right)D_{T}\right){\hat{L}}_{s}P-rs\partial_{X}\left(v_{0}P\right). \end{array}$$ (14)

This is equivalent to a system of Langevin equations. Considering *dr*/*df* = *r*(1 − *r*) the internal variable dynamics (the first term on the right) gives Eq (3) which defines *t*_*E*_. The angular dynamics (the second term on the right) defines *t*_*D*_.

Using the time scale definitions in Eq (4) and non-dimensionalizing time *τ* = *t*/*t*_*M*_ and position *x* = *X*/(*v*_0_*t*_*M*_), we obtain the Fokker-Planck Eq (5).

### Derivation of the drift speed *V*_*D*_, Eq (6), for a log-sensing organism moving in an exponential gradient

From the Fokker-Planck Eq (5) we consider the steady state so that ∂_*τ*_ = 0. For a log-sensing organism moving in an exponential gradient *τ*_*E*_ does not depend on *x*. We can therefore integrate over *x* to get an equation for the marginal steady state distribution $\bar{P}\left(f,s\right)$—this removes the ∂_*x*_ term. Integrating over *s* gives

$$0=-\partial_{f}\left(-\left(f-f_{0}\right)\int \bar{P}w\left(s\right)ds+\frac{r(f)}{\tau_{E}}\int s\bar{P}w\left(s\right)ds\right),$$ (15)

where the bar indicates steady state. By the boundary conditions that *P* → 0 at ±∞, we must have

$$\begin{array}{c} r\left(f\right)\int s\bar{P}w\left(s\right)ds=\tau_{E}\left(f-f_{0}\right)\int \bar{P}w\left(s\right)ds. \end{array}$$ (16)

From the −∂_*x*_(*rsP*) term of the Fokker-Planck Eq (5), the spatial flux is *r*(*f*)*s* and the drift speed is its average over the distribution. Thus we get the drift speed as Eq (6)

$$V_{D}=\bar{\left\langle rs\right\rangle }=\int \int \;r\left(f\right)s\bar{P}w\left(s\right)dsdf=\tau_{E}\;\int \int \;\left(f-f_{0}\right)\bar{P}w\left(s\right)dsdf=\tau_{E}\bar{\left\langle f-f_{0}\right\rangle }.$$ (17)

### Derivation of the analytical solution to the Fokker-Planck Eq (5) by angular moment expansion when *τ*_*D*0_ ≪ 1

Here we use separation of variables and expand the solution to the Fokker-Planck Eq (5) as a sum of eigenfunctions of the operator ${\hat{L}}_{s}$ on *s*. We then ignore high order terms assuming *τ*_*D*0_ ≪ 1 and derive an approximate analytical solution.

The eigenvalue problem of the angular operator ${\hat{L}}_{s}$, defined in Eq (12), is

$$\begin{array}{c} \left(1-s^{2}\right)y^{\prime \prime }-\left(n-1\right)sy^{\prime }=\lambda y. \end{array}$$ (18)

We identify this as the Gegenbauer differential equation [47], with eigenfunctions the Gegenbauer polynomials $C_{k}^{\left(n/2-1\right)}\left(s\right)$ and the corresponding eigenvalues $\lambda_{k}^{\left(n/2-1\right)}=-k\left(k+n-2\right)$. When *n* = 3 they are Legendre polynomials with eigenvalues $\lambda_{k}^{\left(1/2\right)}=-k(k+1)$. The first few Gegenbauer polynomials are

$$\begin{array}{cc} C_{0}^{\left(n/2-1\right)}\left(s\right) & =1\\ C_{1}^{\left(n/2-1\right)}\left(s\right) & =\left(n-2\right)s\\ C_{2}^{\left(n/2-1\right)}\left(s\right) & =\frac{n-2}{2}\left(ns^{2}-1\right). \end{array}$$ (19)

They are orthogonal in the sense that

$$\int_{-1}^{1}C_{k}^{\left(n/2-1\right)}(s)C_{l}^{\left(n/2-1\right)}(s){\left(1-s^{2}\right)}^{\frac{n-3}{2}}\text{d}s=N_{k}^{\left(n/2-1\right)},$$ (20)

where the normalization constants are $N_{k}^{\left(n/2-1\right)}=\frac{\pi 2^{4-n}\left(k+n-3\right)!}{k!\left(2k+n-2\right){\left(\Gamma (n/2-1)\right)}^{2}}$. When *n* = 3 they are $N_{k}^{\left(1/2\right)}=\frac{2}{2k+1}$, those of Legendre polynomials.

The weight in the integration above is consistent with the geometry on an (*n* − 1)-sphere *S*^*n*−1^, whose the volume element are iteratively defined [46] as

$$\begin{array}{c} d_{S^{n-1}}\omega ={\left(\sin\theta \right)}^{n-2}d\theta d_{S^{n-2}}\omega . \end{array}$$ (21)

After a change of variable *s* = cos *θ* and integrating over all remaining dimensions, we see that any integration of *s* should carry a weight

$$\begin{array}{c} w\left(s\right)\text{ds}={\left(1-s^{2}\right)}^{\frac{n-3}{2}}ds. \end{array}$$ (22)

From orthogonality and completeness, we write any function of *s*, in particular the probability distribution *P*, as a series of Gegenbauer polynomials. When *n* = 3 this is the Fourier-Legendre Series.

$$\begin{array}{cc} P(x,f,s,\tau )\; & =\sum_{k=0}^{\infty }p_{k}\left(x,f,\tau \right)\frac{C_{k}^{\left(n/2-1\right)}\left(s\right)}{\sqrt{N_{0}^{\left(n/2-1\right)}N_{k}^{\left(n/2-1\right)}}}\\ & =\frac{1}{N_{0}^{\left(n/2-1\right)}}\left(p_{0}+p_{1}\sqrt{n}s+p_{2}\sqrt{\frac{n+2}{n-1}}\frac{ns^{2}-1}{2}+\cdots \right),\\ p_{k}\left(x,f,\tau \right)\; & =\int_{-1}^{1}\sqrt{\frac{N_{0}^{\left(n/2-1\right)}}{N_{k}^{\left(n/2-1\right)}}}C_{k}^{\left(n/2-1\right)}\left(s\right)P\left(x,f,s,\tau \right){\left(1-s^{2}\right)}^{\frac{n-3}{2}}ds, \end{array}$$ (23)

where we normalize the definitions to ensure $p_{0}=\int_{-1}^{1}P{\left(1-s^{2}\right)}^{\frac{n-3}{2}}ds$ is the same as the marginal distribution. When *n* = 3, the above is

$$\begin{array}{cc} P(x,f,s,\tau )\; & =\sum_{k=0}^{\infty }p_{k}\left(x,f,\tau \right)\frac{\sqrt{2k+1}}{2}C_{k}^{\left(1/2\right)}\left(s\right)\\ & =\frac{1}{2}\left(p_{0}+p_{1}\sqrt{3}s+p_{2}\sqrt{\frac{5}{2}}\frac{3s^{2}-1}{2}+\cdots \right),\\ p_{k}\left(x,f,\tau \right)\; & =\int_{-1}^{1}\sqrt{2k+1}C_{k}^{\left(1/2\right)}\left(s\right)P\left(x,f,s,\tau \right)ds. \end{array}$$ (24)

From now on we denote the marginal distribution *p*(*f*) = *p*_0_(*f*). Also, from this definition $p_{1}=\sqrt{n}\int_{-1}^{1}sP{\left(1-s^{2}\right)}^{\frac{n-3}{2}}ds$.

Substitute the expansion Eq (23) into the Fokker-Planck Eq (5) and use the orthogonality Eq (20), we obtain

$$\partial_{\tau }p_{k}=-\partial_{f}\left(-\left(f-f_{0}\right)p_{k}+\frac{r(f)}{\tau_{E}}{\hat{s}}_{kl}p_{l}\right)+\frac{\lambda_{k}^{\left(n/2-1\right)}}{\left(n-1\right)\tau_{D}\left(f\right)}p_{k}-\partial_{x}\left({\hat{s}}_{kl}p_{l}\right),$$ (25)

where ${\hat{s}}_{kl}=\sqrt{\frac{k(k+n-3)}{\left(2k+n-4)(2k+n-2\right)}}\delta_{k-1,l}+\sqrt{\frac{\left(k+1)(k+n-2\right)}{\left(2k+n-2)(2k+n\right)}}\delta_{k+1,l}$ (summation over *l* implied) is an operator relating neighboring orders. It comes from the positive feedback term. When *n* = 3 it is ${\hat{s}}_{kl}=\frac{k}{\sqrt{4k^{2}-1}}\delta_{k-1,l}+\frac{k+1}{\sqrt{4{\left(k+1\right)}^{2}-1}}\delta_{k+1,l}$. The first few equations are

$$\begin{array}{cc} \partial_{\tau }p= & -\partial_{f}\left(-\left(f-f_{0}\right)p+\frac{r(f)}{\tau_{E}}\frac{1}{\sqrt{n}}p_{1}\right)-r\left(f\right)\partial_{x}\frac{1}{\sqrt{n}}p_{1}\\ \partial_{\tau }p_{1}= & -\partial_{f}\left(-\left(f-f_{0}\right)p_{1}+\frac{r(f)}{\tau_{E}}\left(\frac{1}{\sqrt{n}}p+\sqrt{\frac{2(n-1)}{n\left(n+2\right)}}p_{2}\right)\right)\\ & -\frac{1}{\tau_{D}\left(f\right)}p_{1}-r\left(f\right)\partial_{x}\left(\frac{1}{\sqrt{n}}p+\sqrt{\frac{2(n-1)}{n\left(n+2\right)}}p_{2}\right)\\ \partial_{\tau }p_{2}= & -\partial_{f}\left(-\left(f-f_{0}\right)p_{2}+\frac{r(f)}{\tau_{E}}\left(\sqrt{\frac{2(n-1)}{n\left(n+2\right)}}p_{1}+\sqrt{\frac{3n}{\left(n+2\right)\left(n+4\right)}}p_{3}\right)\right)\\ & -\frac{2n}{\left(n-1\right)\tau_{D}\left(f\right)}p_{2}-r\left(f\right)\partial_{x}\left(\sqrt{\frac{2(n-1)}{n\left(n+2\right)}}p_{1}+\sqrt{\frac{3n}{\left(n+2\right)\left(n+4\right)}}p_{3}\right). \end{array}$$ (26)

In the definition of ${\hat{s}}_{kl}$, when *k* ≫ 1 the non-zero entries approach a constant 1/2. This means for large *k* the coefficients *p*_*k*_ in Eq (25) evolve similarly except that higher orders decay with faster rates *k*(*k* + *n* − 2)/(*n* − 1)*τ*_*D*_. Therefore when *τ*_*D*0_ ≪ 1 we can neglect the 2nd and higher orders, which closes the infinite series of moment equations and leaves two equations concerning the zeroth and first marginal moments in *s*, *p*(*x*, *f*, *τ*) and *p*_1_(*x*, *f*, *τ*) respectively. At steady state the approximation gives the analytical solution

$$\bar{p}\left(f\right)=\frac{1}{W}\frac{\frac{r(f)}{\tau_{E}}}{\frac{1}{n}{\left(\frac{r(f)}{\tau_{E}}\right)}^{2}-{\left(f-f_{0}\right)}^{2}}\exp\left(-\int^{f}\frac{\frac{f_{1}-f_{0}}{\tau_{D}\left(f_{1}\right)}}{\frac{1}{n}{\left(\frac{r(f_{1})}{\tau_{E}}\right)}^{2}-{\left(f_{1}-f_{0}\right)}^{2}}df_{1}\right),$$ (27)

where *W* is a normalization constant. Eq (27) is the same as Eq (7) in the main text.

We can interpret the steady state distribution as a potential solution $\bar{p}\left(f\right)\propto \exp(-V\left(f)\right)$ where *V*(*f*) is the “potential”. In this case the equivalent “force” in internal state is

$$\begin{array}{cc} F(f)= & -V^{\prime }\left(f\right)=\frac{d\ln\bar{p}\left(f\right)}{df}\\ = & \frac{d}{df}\ln\frac{\frac{r(f)}{\tau_{E}}}{\frac{1}{n}{\left(\frac{r(f)}{\tau_{E}}\right)}^{2}-{\left(f-f_{0}\right)}^{2}}-\frac{\frac{f-f_{0}}{\tau_{D}\left(f\right)}}{\frac{1}{n}{\left(\frac{r(f)}{\tau_{E}}\right)}^{2}-{\left(f-f_{0}\right)}^{2}}. \end{array}$$ (28)

Since *τ*_*D*0_ ≪ 1 the second term dominates, making the “force” a spring-like system, with spring constant

$$k\left(f\right)=\frac{\frac{1}{\tau_{D}\left(f\right)}}{\frac{1}{n}{\left(\frac{r(f)}{\tau_{E}}\right)}^{2}-{\left(f-f_{0}\right)}^{2}}.$$ (29)

Three observations can be made from this spring constant in intuitively understanding the steady state distribution $\bar{p}\left(f\right)$. (i) *k*(*f*)→∞, i.e. the “spring” becomes infinitely “stiff”, when the denominator approaches 0. Therefore, the bounds of the distribution $\bar{p}\left(f\right)$ are proportional to 1/*τ*_*E*_, the ratio between the positive and negative feedbacks (Eq (4)). Intuitively, a stronger positive feedback (smaller *τ*_*E*_) drives the internal state *f* further away from *f*_0_, so the spring constant *k*(*f*) is smaller and the distribution $\bar{p}\left(f\right)$ is wider. (ii) A slower change in direction (smaller *τ*_*D*_) leads to a larger spring constant *k*(*f*) ∝ 1/*τ*_*D*_(*f*), and thus the distribution $\bar{p}\left(f\right)$ is more concentrated near the “origin” *f*_0_. Intuitively, a shorter direction correlation time *τ*_*D*_ inhibits coherent motion in a single direction, which is required by the positive feedback to consistently drive the internal state *f* away. Thus the distribution $\bar{p}\left(f\right)$ is more concentrated. (iii) Asymmetries are created by the functional dependencies of *r*(*f*) and *τ*_*D*_(*f*), both increasing in *f*—a “weaker spring” for higher values of *f* shifts the distribution $\bar{p}\left(f\right)$ there. Intuitively, more positive feedback ∝ *r*(*f*) and more coherent motion ∝ *τ*_*D*_(*f*) in the positive direction asymmetrically drives the internal state towards higher values. These 3 observations can all be found in Fig 1C.

### Derivation of the distribution $\bar{p}\left(f\right)$ and drift speed *V*_*D*_ when the negative feedback dominates

We expand the steady state solution Eq (27) in orders of $\frac{1}{\tau_{E}}⪡1$ and *τ*_*D*0_ ≪ 1 and obtain a near-Gaussian approximation, from which we integrate using Eq (6) to obtain MFT results.

First, we write the steady state distribution Eq (27) as

$$\begin{array}{c} \bar{p(f)}=\frac{1}{W}B\left(f\right)\exp\left(-\int^{f}A\left(f_{1}\right)df_{1}\right). \end{array}$$ (30)

From the Taylor expansion of the integrand in the exponent

$$\begin{array}{cc} A(f)= & \frac{\frac{f-f_{0}}{\tau_{D}\left(f\right)}}{\frac{1}{n}{\left(\frac{r(f)}{\tau_{E}}\right)}^{2}-{\left(f-f_{0}\right)}^{2}}\\ = & \frac{n\tau_{E}^{2}}{r_{0}^{2}\tau_{D0}}\left(f-f_{0}\right)+\left(1+O(\frac{1}{\tau_{E}^{2}})\right)\Sigma_{m=1}^{\infty }\frac{n^{m+1}\tau_{E}^{2m+2}}{r_{0}^{2m+2}\tau_{D0}}{\left(f-f_{0}\right)}^{2m+1}\\ & -\left(1+O(\frac{1}{\tau_{E}^{2}})\right)\Sigma_{m=1}^{\infty }\frac{n^{m}\tau_{E}^{2m}r_{0}^{\prime }}{r_{0}^{2m+1}\tau_{D0}}\left(2m+\frac{r_{0}\tau_{D0}^{\prime }}{r_{0}^{\prime }\tau_{D0}}\right){\left(f-f_{0}\right)}^{2m}, \end{array}$$ (31)

where ′ = *d*/*df*, we see that if we define

$$\sigma^{2}=\frac{r_{0}^{2}\tau_{D0}}{n\tau_{E}^{2}},$$ (32)

the first term in *A*(*f*) will give $-\int A\left(f\right)df=-\frac{{\left(f-f_{0}\right)}^{2}}{2\sigma^{2}}+...$. If we can show that the rest of the terms are small when $\frac{1}{\tau_{E}}<1$ and *τ*_*D*0_ < 1, we can write $\bar{p}\left(f\right)$ as a small deviation from a Gaussian.

Indeed, if we consider the integration range $|f-f_{0}|∼\sigma ∼O\left(\sqrt{\tau_{D0}}/\tau_{E}\right)$, we can write

$$\begin{array}{cc} -\int_{f_{0}}^{f}A\left(f_{1}\right)df_{1}= & -\frac{{\left(f-f_{0}\right)}^{2}}{2\sigma^{2}}-\Sigma_{m=1}^{\infty }\frac{\tau_{D0}^{m}}{2m+2}\frac{{\left(f-f_{0}\right)}^{2m+2}}{\sigma^{2m+2}}\\ & +\Sigma_{m=1}^{\infty }\frac{r_{0}^{\prime }}{r_{0}}\frac{\tau_{D0}^{m-1}}{2m+1}\left(2m+\frac{r_{0}\tau_{D0}^{\prime }}{r_{0}^{\prime }\tau_{D0}}\right)\frac{{\left(f-f_{0}\right)}^{2m+1}}{\sigma^{2m}}+O\left(\frac{1}{\tau_{E}^{2}}\right)\\ = & -\frac{{\left(f-f_{0}\right)}^{2}}{2\sigma^{2}}+\frac{r_{0}^{\prime }}{r_{0}}\frac{1}{3}\left(2+\frac{r_{0}\tau_{D0}^{\prime }}{r_{0}^{\prime }\tau_{D0}}\right)\frac{{\left(f-f_{0}\right)}^{3}}{\sigma^{2}}-\frac{\tau_{D0}}{4}\frac{{\left(f-f_{0}\right)}^{4}}{\sigma^{4}}\\ & +\frac{r_{0}^{\prime }}{r_{0}}\frac{\tau_{D0}}{5}\left(4+\frac{r_{0}\tau_{D0}^{\prime }}{r_{0}^{\prime }\tau_{D0}}\right)\frac{{\left(f-f_{0}\right)}^{5}}{\sigma^{4}}+O\left(\frac{1}{\tau_{E}^{2}}\right)+O\left(\tau_{D0}^{2}\right), \end{array}$$ (33)

Similarly, the prefactor is

$$B(f)=\frac{n\tau_{E}}{r_{0}}(1-\frac{r_{0}^{\prime }}{r_{0}}\left(f-f_{0}\right)+\tau_{D0}\frac{{\left(f-f_{0}\right)}^{2}}{\sigma^{2}}-3\tau_{D0}\frac{r_{0}^{\prime }}{r_{0}}\frac{{\left(f-f_{0}\right)}^{3}}{\sigma^{2}}+O\left(\frac{1}{\tau_{E}^{2}}\right)+O\left(\tau_{D0}^{2}\right)).$$ (34)

Substitute Eqs (33) and (34) back into Eq (30) and taking care of the orders of all cross terms, we obtain

$$\begin{array}{cc} \bar{p}\left(f\right) & =\frac{1}{Z}\frac{e^{-\frac{{\left(f-f_{0}\right)}^{2}}{2\sigma^{2}}}}{\sqrt{2\pi \sigma^{2}}}(1-\frac{r_{0}^{\prime }}{r_{0}}\left(f-f_{0}\right)+\tau_{D0}\frac{{\left(f-f_{0}\right)}^{2}}{\sigma^{2}}\\ & +\frac{r_{0}^{\prime }}{3r_{0}}\left(2-9\tau_{D0}+\frac{r_{0}\tau_{D0}^{\prime }}{r_{0}^{\prime }\tau_{D0}}\right)\frac{{\left(f-f_{0}\right)}^{3}}{\sigma^{2}}-\frac{\tau_{D0}}{4}\frac{{\left(f-f_{0}\right)}^{4}}{\sigma^{4}}\\ & +\frac{r_{0}^{\prime }}{60r_{0}}\left(103+32\frac{r_{0}\tau_{D0}^{\prime }}{r_{0}^{\prime }\tau_{D0}}\right)\tau_{D0}\frac{{\left(f-f_{0}\right)}^{5}}{\sigma^{4}}\\ & -\frac{r_{0}^{\prime }}{12r_{0}}\left(2+\frac{r_{0}\tau_{D0}^{\prime }}{r_{0}^{\prime }\tau_{D0}}\right)\tau_{D0}\frac{{\left(f-f_{0}\right)}^{7}}{\sigma^{6}}+O\left(\frac{1}{\tau_{E}^{3}}\right)+O\left(\tau_{D0}^{\frac{5}{2}}\right)). \end{array}$$ (35)

with normalization constant *Z*.

We notice from Eq (30) that the range of distribution is bounded by *f*_*L*_ and *f*_*U*_, defined by

$$\begin{array}{cc} f_{L}-f_{0}= & -\frac{1}{\sqrt{n}}\frac{r(f_{L})}{\tau_{E}},\\ f_{U}-f_{0}= & \frac{1}{\sqrt{n}}\frac{r(f_{U})}{\tau_{E}}. \end{array}$$ (36)

Since $\sigma =\frac{r_{0}\sqrt{\tau_{D0}}}{\sqrt{n}\tau_{E}}⪡\frac{r_{0}}{\sqrt{n}\tau_{E}}$, we see that the integration range is much larger than the standard deviation of the Gaussian factor, and thus can be considered from −∞ to ∞. Therefore we get the normalization constant

$$Z=1+\frac{\tau_{D0}}{4}+O\left(\frac{1}{\tau_{E}^{2}}\right)+O\left(\tau_{D0}^{2}\right).$$ (37)

Substitute Eqs (35) and (37) into Eq (6) and carry out the integrals

$$V_{D}=\frac{r_{0}\tau_{D0}}{n\tau_{E}}\frac{\left(1-\frac{3}{4}\tau_{D0}\right)\left(r_{0}^{\prime }+r_{0}\frac{\tau_{D0}^{\prime }}{\tau_{D0}}\right)+O\left(\frac{1}{\tau_{E}}\right)+O\left(\tau_{D0}^{\frac{3}{2}}\right)}{1+\frac{\tau_{D0}}{4}+O\left(\frac{1}{\tau_{E}^{2}}\right)+O\left(\tau_{D0}^{2}\right)}.$$ (38)

Finally, noticing that by the definition of *τ*_*D*_ in Eq (4)

$$\tau_{D}\left(f\right)=\tau_{D0}\frac{r_{0}D_{R}+\left(1-r_{0}\right)D_{T}}{r\left(f\right)D_{R}+\left(1-r(f)\right)D_{T}},$$ (39)

we can get

$$\tau_{D0}^{\prime }=\tau_{D0}\frac{D_{T}-D_{R}}{r_{0}D_{R}+\left(1-r_{0}\right)D_{T}}r_{0}^{\prime }.$$ (40)

Therefore

$$\begin{array}{cc} r_{0}^{\prime }+r_{0}\frac{\tau_{D0}^{\prime }}{\tau_{D0}} & =\frac{\tau_{D0}^{\prime }}{\tau_{D0}}\left(\frac{r_{0}D_{R}+\left(1-r_{0}\right)D_{T}}{D_{T}-D_{R}}+r_{0}\right)\\ & =\frac{\tau_{D0}^{\prime }}{\tau_{D0}}\frac{D_{T}}{D_{T}-D_{R}}. \end{array}$$ (41)

Taking *D*_*T*_ ≫ *D*_*R*_, we put this back into Eq (38) and get

$$\begin{array}{cc} V_{D} & =\frac{r_{0}\tau_{D0}^{\prime }}{n\tau_{E}}\frac{1-\frac{3}{4}\tau_{D0}+O\left(\frac{1}{\tau_{E}}\right)+O\left(\tau_{D0}^{\frac{3}{2}}\right)}{1+\frac{\tau_{D0}}{4}+O\left(\frac{1}{\tau_{E}^{2}}\right)+O\left(\tau_{D0}^{2}\right)}\\ & =\frac{r_{0}\tau_{D0}^{\prime }}{n\tau_{E}\left(1+\tau_{D0}\right)}\left(1+O\left(\frac{1}{\tau_{E}}\right)+O\left(\tau_{D0}^{\frac{3}{2}}\right)\right). \end{array}$$ (42)

When converted back to real units (*t* instead of *τ* = *t*/*t*_*M*_), the highest-order term is identical, except for notations, to Eq (3) in Dufour *et al.* [13] obtained from a different approach. It can also be reduced to Eq (12) in Si *et al.* [12] by assuming a high running probability and a long memory. It agrees with Eq (6.24) in Erban & Othmer [48] and Eq (16) in Franz *et al.* [49] with appropriate inclusion of rotational diffusion.

In Eq (38) we expanded the distribution as a near-Gaussian around *f*_0_. From Eq (6) we see the mean internal state $f_{m}=\bar{\langle f\rangle }$ has a slight shift, so it’s more accurate to expand around *f*_*m*_. From Eqs (38) and (6) in the main text, we see $\bar{\langle f-f_{0}\rangle }∼O\left(1/\tau_{E}^{2}\right)$. Thus considering the shift in *f*_*m*_ the resulting *V*_*D*_ has the same form compared to Eq (42):

$$V_{D}=\frac{r_{m}\tau_{Dm}^{\prime }}{n\tau_{E}\left(1+\tau_{Dm}\right)}\left(1+O\left(\frac{1}{\tau_{E}}\right)+O\left(\tau_{Dm}^{\frac{3}{2}}\right)\right).$$ (43)

### Bounds of the distribution *p*(*f*)

The first term in Eq (5) says the flux in *f*-space is non-negative provided −(*f* − *f*_0_) + *rs*/*τ*_*E*_ > 0, or, noting *s* ≤ 1

$$f\leq f_{0}+r\left(f\right)s/\tau_{E}\leq f_{0}+r\left(f\right)/\tau_{E}.$$ (44)

Thus the upper bound *f*_*U*_ of the distribution *p*(*f*) is achieved at equality. Similarly, the lower bound *f*_*L*_ is achieved when we take equal signs of

$$f\geq f_{0}+r\left(f\right)s/\tau_{E}\geq f_{0}-r\left(f\right)/\tau_{E},$$ (45)

noting *s* ≥ 1.

When *τ*_*E*_ becomes small we note *f*_*U*,*L*_ deviates far away from *f*_0_ as 1/*τ*_*E*_ → ∞. Using the definition *r* = 1/(1 + exp(−*f*)), we write

$$\begin{array}{c} f_{L,U}=\mp \frac{1}{\tau_{E}\left(1+\exp\left(-f_{L,U}\right)\right)}. \end{array}$$ (46)

The plus sign gives exp(−*f*_*U*_) ≪ 1 and *f*_*U*_ ≈ 1/*τ*_*E*_. The minus sign gives exp(−*f*_*L*_) ≫ 1 and *f*_*L*_ = −exp(*f*_*L*_)/*τ*_*E*_. Taking logarithm, the latter gives *f*_*L*_ = ln (|*f*_*L*_|*τ*_*E*_) ≈ ln *τ*_*E*_.

### Derivation of Langevin Eq (8)

To derive Langevin equations from the Fokker-Planck equation we need to consider the geometric weight factor *w*(*s*) in Eq (22) for anglular integration. In deriving *s*-dynamics, we start with the angular part of the Fokker-Planck Eq (5)

$$\partial_{\tau }P=\frac{{\hat{L}}_{s}P}{\left(n-1\right)\tau_{D}\left(f\right)}+\ldots .$$ (47)

Multiplying an arbitrary function *A*(*s*) and integrating over all dimensions, we obtain

$$\begin{array}{cc} & \int dx\int df\int_{-1}^{1}w\left(s\right)dsA\left(s\right)\partial_{\tau }P\\ = & \int dx\int df\int_{-1}^{1}w\left(s\right)dsA\left(s\right)\frac{{\left(1-s^{2}\right)}^{\frac{3-n}{2}}\partial_{s}\left({\left(1-s^{2}\right)}^{\frac{n-1}{2}}\partial_{s}P\right)}{\left(n-1\right)\tau_{D}\left(f\right)}\\ = & -\int dx\int df\int_{-1}^{1}w\left(s\right)ds\frac{sP}{\tau_{D}\left(f\right)}\partial_{s}A\left(s\right)\\ & +\int dx\int df\int_{-1}^{1}w\left(s\right)ds\frac{\left(1-s^{2}\right)P}{\left(n-1\right)\tau_{D}\left(f\right)}\partial_{s}^{2}A\left(s\right). \end{array}$$ (48)

To apply the standard result of equivalence between Fokker-Planck equations and Langevin equations, we need to change the measure in *s*-space to unity. This prompts the definition *Q*(*s*, *t*) = *w*(*s*)∬*P*(*y*, *f*, *s*, *t*)d*x*d*f* so that the above becomes

$$\begin{array}{cc} \int_{-1}^{1}dsA\left(s\right)\partial_{\tau }Q= & -\int_{-1}^{1}ds\frac{sQ}{\tau_{D}\left(f\right)}\partial_{s}A\left(s\right)+\int_{-1}^{1}ds\frac{\left(1-s^{2}\right)Q}{\left(n-1\right)\tau_{D}\left(f\right)}\partial_{s}^{2}A\left(s\right)\\ = & \int_{-1}^{1}dsA\left(s\right)\partial_{s}\frac{sQ}{\tau_{D}\left(f\right)}+\int_{-1}^{1}dsA\left(s\right)\partial_{s}^{2}\frac{\left(1-s^{2}\right)Q}{\left(n-1\right)\tau_{D}\left(f\right)}, \end{array}$$ (49)

where we integrated by parts and discarded boundary terms. Since *A*(*s*) is an arbitrary function, we can write down the Fokker-Planck equation

$$\partial_{\tau }Q=\partial_{s}\frac{sQ}{\tau_{D}\left(f\right)}+\partial_{s}^{2}\frac{\left(1-s^{2}\right)Q}{\left(n-1\right)\tau_{D}\left(f\right)},$$ (50)

which is equivalent [45] to the Langevin equation

$$\frac{ds}{d\tau }=-\frac{s}{\tau_{D}\left(r\right)}+\sqrt{\frac{2\left(1-s^{2}\right)}{\left(n-1\right)\tau_{D}\left(r\right)}}\eta \left(\tau \right).$$ (51)

where *η*(*τ*) denotes the Gaussian white noise with 〈*η*(*τ*_1_)*η*(*τ*_2_)〉 = *δ*(*τ*_1_ − *τ*_2_).

The other two variables follow standard results [45] from the Fokker-Planck Eq (5) in the main text

$$\begin{array}{cc} \frac{df}{d\tau } & =-\left(f-f_{0}\right)+\frac{r(f)s}{\tau_{E}},\\ \frac{dx}{d\tau } & =r(f)s. \end{array}$$ (52)

Now we change variables according to the definitions *r*(*f*) = 1/(1 + exp(−*f*)) and *v* = *rs*, and derive from the above dynamics in Eqs (51) and (52) to get the Langevin Eq (8).

### Linear response near the fixed point of the Langevin system

Near the fixed point (*r*_0_, 0), the eigenvectors and eigenvalues of the linearized Langevin Eq (8) are:

$$\begin{array}{cc} \left[\begin{array}{c} 1\\ 0 \end{array}\right] & \;\;\;\text{for}\;\text{eigenvalue}\;\;\;-1;\\ \left[\begin{array}{c} \frac{\left(1-r_{0}\right)r_{0}}{\tau_{E}}\frac{\tau_{D0}}{\tau_{D0}-1}\\ 1 \end{array}\right] & \;\;\;\text{for}\;\text{eigenvalue}\;\;\;-\frac{1}{\tau_{D0}}. \end{array}$$ (53)

When *τ*_*E*_ is large, $\frac{(1-r_{0})r_{0}}{\tau_{E}}\frac{\tau_{D0}}{\tau_{D0}-1}⪡1$ and the eigenvectors are almost orthogonal. When *τ*_*E*_ is small, $\frac{(1-r_{0})r_{0}}{\tau_{E}}\frac{\tau_{D0}}{\tau_{D0}-1}\gg 1$ and the eigenvectors are not orthogonal.

### Numerical methods

In Fig 1A heat map the drift speed *V*_*D*_ was calculated by fitting the linear part of the mean trajectory. In Fig 1B the first 50 *s* were removed to avoid the start up transient. In Fig 1C, the steady state $\bar{p}\left(f\right)$ from agent-based simulations was calculated from the histogram of all the internal values of the 10^4^ simulated cells between *τ* = 10 and *τ* = 20, sampled at regular steps of *τ* = 0.01. Numerical solutions of the Fokker-Planck Eq (5) were obtained by expanding the distribution in angles, as in Eq (25), and keeping the first 10 orders. The steady state $\bar{p}\left(f\right)$ was found by solving an initial value problem using the NDSolve function in Mathematica, with 10^4^ spatial points and integration time up to *τ* = 10. Further orders, finer grid, and longer integration times were checked to ensure solution accuracy. In Fig 1D, *V*_*D*_ from agent-based and Fokker-Planck were calculated by plugging into Eq (6) $\bar{p}\left(f\right)$ obtained from those methods in C. MFT was calculated by combining Eq (42) with Eq (6) to find both $f_{m}=\bar{\langle f\rangle }$ and *V*_*D*_ [12, 13]. In the inset, the black curves show the approximate distribution in Eq (35).

In Fig 2B and 2D the Langevin trajectories were generated using Euler’s method to integrate Eq (8).

In Fig 3C, 3F and 3I the *τ*_*E*_ calculation considered receptor saturation as well as the varying gradient length scales, with *C* and *L* evaluated at mean positions. Note this is not the average *τ*_*E*_ over the population.

## Supporting information

S1 Appendix Agent-based Models and Numerical Methods. (PDF)

S1 FigRobustness of results.(A) Same as Fig 1A (where *r*_0_ = 0.8, Table S1 Table) except with *r*_0_ = 0.5. (B) *r*_0_ = 0.7. (C) Same as Fig 1A (where *D*_*T*_/*D*_*R*_ ≈ 37, Table S1 Table) except with *D*_*T*_/*D*_*R*_ = 5. (D) Same as Fig 1A except without assuming receptors in log-sensing range, i.e. Eq (S7) was used rather than Eq (S11). (E) Same as D, but additionally implements adaptation asymmetry, where the adaptation rate $t_{M}^{-1}$ in equation Eq (S4) depends on *m* [42]. Here the adaptation is 3 times faster when *m* > *m*(*C*) than when *m* < *m*(*C*), and *t*_*M*_ is defined as the time scale when *m* < *m*(*C*). (F) Same as D but with nonlinear adaptation rate Eq (S4). Values for the parameters are: *a*_*B*_ = 0.74, *r*_*B*_ = 4.0, *K*_*R*_ = 0.32, *K*_*B*_ = 0.30, and *V*_*R*_ and *V*_*B*_(0) chosen to ensure $\frac{dm}{dt}=0$ when *a* = *a*_0_ and the adaptation time is *t*_*M*_ when linearized.(TIF)

S2 Fig Effect of changing *τ*_*D*_. 5 sample trajectories (thin solid curves) and the mean over 10^4^ sample trajectories (thick lines) of non-dimensionalized position *x* = *X*/(*v*_0_*t*_*M*_) as a function of time *τ* = *t*/*t*_*M*_. Colors correspond to cells with matching (green *τ*_*D*0_ = 1) and non-matching (orange *τ*_*D*0_ = 0.1) reorientation as in Fig 1 (same methods).
(TIF)

S3 FigEnhanced chemotaxis with signaling noise.(A) Same as Fig 1A but adding a signaling noise term $\sigma_{m}\sqrt{2/t_{M}}\Gamma \left(t\right)$ in Eq (S6) where Γ(*t*) is the standard Wiener process (see Eq [4] in [20]). From Eqs (S1)-(S2) and *Y* = *αa* we obtain *dY*/*dm* = *Y*(1 − *a*)*ϵ*_1_. Then plugging in *σ*_*Y*_/*Y* = 0.1 [20, 21], *a* ≈ 0.5 and *ϵ*_1_ = −1, we obtain *σ*_*m*_ = 0.2. (B) The absolute difference in drift speed between A (with signaling noise) and Fig 1A (without signaling noise), Δ*V*_*D*_ = *V*_*D*_|_*noise*_ − *V*_*D*_|_*nonoise*_, shows how signaling noise can either enhance or reduce the drift speed depending on (*τ*_*E*_, *τ*_*D*_). Note the colorbar is different. Black contour lines show level sets of Δ*V*_*D*_ at the colorbar ticks (-0.05, -0.025, 0, 0.025, and 0.05). (C) The relative difference in drift speed (B divided by the drift speed without signaling noise). Again, the color scale is different and black contour lines show level sets at the colorbar ticks.(TIF)

S1 Table Parameter values used in agent-based simulations. (PDF)

S1 Movie Movie of the (*r*, *v*) phase space trajectories shown in Fig 2. (AVI)
