# Supplementary material for: Feedback between motion and sensation provides nonlinear boost in run-and-tumble navigation
Source: PLoS Comput Biol. 2017 Mar 6;13(3):e1005429. doi: 10.1371/journal.pcbi.1005429 (PMC5358899; doi:10.1371/journal.pcbi.1005429)
Supplement: S1 Appendix — (PDF) [file pcbi.1005429.s001.pdf]

S1 Appendix. Agent-based Models and Numerical Methods

Model of the chemotaxis pathway

We used a standard model of *E. coli* chemotaxis [1–5] in which the cell relays information from the external environment to the flagellar motor through a signaling cascade triggered by the binding of ligand to transmembrane chemoreceptors (parameters in S1 Table). The receptors form cooperative clusters, the activity of which is described by a two-state model where the activity a is determined by the free energy difference F (in units of $k_B T$) between the active and inactive states

$$a = \frac{1}{1 + \mathrm{e}^F}. \tag{S1}$$

Two terms, $F = F_m(m) + F_C(C)$, contribute to the free energy. The former depends on the methylation level m of the receptor

$$F_m(m) = \epsilon_0 + \epsilon_1 m, \tag{S2}$$

with constants ϵ_0 and ϵ_1, while the latter depends on the ligand concentration C

$$F_C(C) = N^{Rec} \ln \frac{1 + C/K_i}{1 + C/K_a}. \tag{S3}$$

where K_a and K_i are the ligand dissociation constants of the active and inactive states and N^{Rec} is the degree of cooperativity of the cluster, i.e. the average size of the subclusters that switch as all-or-none units within the cluster of receptors.

As the external environment signals changes in $F_C(C)$, the receptor adapts via methylation and demethylation to control $F_m(m)$, trying to maintain an activity level a_0 independent of the environment. The methylation kinetics is described by Eq (4) of [3]

$$\frac{\mathrm{d}m}{\mathrm{d}t} = V_R \frac{1 - a}{K_R + 1 - a} - V_B(a) \frac{a}{K_B + a}, \tag{S4}$$

where V_R and $V_B(a) = V_B(0)\left(1 + \theta(a - a_B)\frac{a - a_B}{1 - a_B} r_B\right)$ (θ is the Heaviside function and $a_B = 0.74$ and $r_B = 4.0$) are the methylation/demethylation rates of the proteins CheR and CheB, and K_R and K_B are their the dissociation constants respectively. Because $\epsilon_1 < 0$ (S1 Table), Eqs (S1)–(S4) show that methylation and demethylation reactions tends to maintain the system at a constant activity level a_0.

Assuming the signal transduction is fast compared to adaptation kinetics, the receptor activity determines the concentration of the response regulator CheY-P, $Y(a) = \alpha a$ with α constant. CheY-P then binds to the flagellar motor complex, modulating its switching between rotating clockwise (CW) or counterclockwise (CCW), which are Poisson processes with rates from CCW to CW as $\lambda_{CCW}(Y)$ and from CW to CCW as $\lambda_{CW}(Y)$

$$\lambda_{CCW,CW}(Y) = \omega \exp\left(\mp G(Y)\right), \quad \text{with} \quad G(Y) = \frac{\epsilon_2}{4} - \frac{\epsilon_3}{2} \frac{1}{1 + K/Y}, \tag{S5}$$

where ω, ϵ_2, ϵ_3, and K are constants.

The motor state of each flagellum determines its conformation to be in one of normal, curly-1, and semi-coiled. The flagellar conformations in turn determine the cell's motility state (run or tumble). For a single-flagellum cell, the motor states CCW and CW corresponds to the motility states run and tumble, and we can write $\lambda_R = \lambda_{CCW}$ and $\lambda_T = \lambda_{CW}$ as the switching rates from run to tumble and from tumble to run, respectively [4].

Analytical approximations

In this section we show how the standard bacterial chemotaxis model just described maps onto the minimal model of run-and-tumble navigation used for analytical derivations in the main text. Doing so requires

making simplifying assumptions. In S1 Fig D,F we verify the validity of these approximations by running agent-based simulations with and without such simplifications.

Following [4] methylation-demethylation kinetics are modeled as a linear relaxation around the adapted level of methylation $m_0(C)$ that maintains a_0. From Eqs (S1)–(S4) we get

$$\frac{\mathrm{d}m}{\mathrm{d}t} = -\frac{m - m_0(C)}{t_M}, \tag{S6}$$

where t_M is the adaptation time and

$$m_0(C) = \frac{1}{\epsilon_1}\left(F_0 - N^{Rec}\ln\frac{1 + C/K_i}{1 + C/K_a} - \epsilon_0\right), \quad \text{with} \quad F_0 = -\ln\left(1/a_0 - 1\right). \tag{S7}$$

so that when $m = m_0(C)$ it follows that $F = F_0$ and $a = a_0$. Substituting in Eq (S6) we obtain:

$$\frac{\mathrm{d}F}{\mathrm{d}t} = -\frac{F - F_0}{t_M} + N^{Rec}\frac{\mathrm{d}}{\mathrm{d}t}\ln\frac{1 + C/K_i}{1 + C/K_a} = -\frac{F - F_0}{t_M} + N^{Rec}\left(\frac{1}{K_i/C + 1} - \frac{1}{K_a/C + 1}\right)\frac{\mathrm{d}}{\mathrm{d}t}\ln C, \tag{S8}$$

where $\mathrm{d}/\mathrm{d}t = \partial/\partial t + \dot{\mathbf{X}}\cdot\nabla$ is the material derivative along the path of the cell. Comparing this equation with Eq (1) in the main text, we define the perceived signal as the log-concentration

$$\phi = \ln\frac{C}{K_i}, \tag{S9}$$

while the receptor gain is concentration-dependent

$$N = N^{Rec}\left(\frac{1}{K_i/C + 1} - \frac{1}{K_a/C + 1}\right). \tag{S10}$$

The concentration-dependent factor is always less than 1, and as C increases this factor contributes to a smaller gain. Within the range of sensitivity of the receptor, i.e. when $K_i \ll C \ll K_a$, this factor is close to 1 and we obtain the log-sensing approximation. In this case, Eq (S7) can be further simplified to

$$m_0(C) \approx \frac{1}{\epsilon_1}\left(F_0 - N^{Rec}\ln\frac{C}{K_i} - \epsilon_0\right), \quad \text{with} \quad F_0 = -\ln\left(1/a_0 - 1\right). \tag{S11}$$

Given constant Poisson switching rates λ_R and λ_T, the probability to be running is determined by $r = \lambda_T/\left(\lambda_R + \lambda_T\right)$. From Eq (S5) and the definition $Y = \alpha a = \alpha/\left(1 + \mathrm{e}^F\right)$ we can write

$$r(F) = \frac{1}{1 + \frac{\lambda_R}{\lambda_T}} = \frac{1}{1 + \exp\left(-\frac{\epsilon_2}{2} + \epsilon_3\frac{1}{1 + \frac{K}{\alpha}\left(1 + \mathrm{e}^F\right)}\right)}. \tag{S12}$$

Thus the probability to run r is a monotonically increasing function of the free energy F. Its shape is almost identical to the standard sigmoidal function $1/\left(1 + \exp\left(-H\left(F - \delta\right)\right)\right)$ with a scaling H and shift δ. Linearly expanding both expressions and matching zeroth and first order we obtain:

$$\begin{aligned} \delta &= -\ln\left(\left(2\epsilon_3/\epsilon_2 - 1\right)\alpha/K - 1\right) \\ H &= \epsilon_3\mathrm{e}^{-\delta}\left(\frac{\epsilon_2}{2\epsilon_3}\right)^2\frac{K}{\alpha}. \end{aligned} \tag{S13}$$

For the parameters chosen we have $\delta \approx 0$ (S1 Table). Therefore, $r = \lambda_T/\left(\lambda_R + \lambda_T\right) \approx 1/\left(1 + \exp\left(-HF\right)\right)$.

Agent-based simulations

The agent-based chemotaxis simulations were performed using Euler's method as described previously [1, 4, 6, 7]. At each time, each cell moves forward or stays in place according to its motility state (run or tumble), which also determines whether its direction changes with rotational diffusion coefficients D_R or

D_T. Once the position, direction and local ligand concentrations are updated, the adaptation equation is integrated and the free energy difference and activity of the receptors is updated. This in turn determines the switching rates according to Eq (S5) and $Y(t) = \alpha a(t)$, and the motor state is changed if a random number drawn exceeds the probability to switch over the time step. After that the motor state determines the flagellum state and subsequently the cell motility state with rules and parameters as in [4], completing one time step.

We used three different types of agent-based simulation:

1. The full nonlinear simulation integrated equations Eqs (S1)–(S5) and served as our reference model of bacterial chemotaxis against which we tested our approximations (S1 Fig F).

2. The second model [1, 4] used linear adaptation kinetics replacing Eq (S4) with Eqs (S6)–(S7). This model was used to generate Fig 3 as well as the scatter plot in Fig 1A. Note that for the latter, the values of the parameters t_M, f_0, v_0, D_R and D_T where chosen according to random distributions to match the phenotypic heterogeneity measured in wild type population of *E. coli* (see below).

3. The third model was the same as the second one with the added simplification that the cell was assumed to be perfectly log sensing: Eq (S11) was used instead of Eq (S7). This model was used to generate the heat map in Fig 1A.

Simulation setup

In Fig 1A heat map, model 3 was used to generate 10^4 sample trajectories that were each 200 seconds long — sufficient for the cells to reach steady state — for each parameter set τ_E and τ_{D0}. We used constant run speed $v_0 = 20 \mu m/s$, memory length $t_M = 10\ s$, and adapted probability to run $r_0 = 0.8$ for all cells and all parameter sets. The gradient length scale L and rotational diffusion coefficients D_R and D_T were then determined by each timescale ratios. We started the cells at the bottom of an exponential gradient where the initial concentration is $C_i = 0.1\ mM$.

In Fig 1A scatter plot, model 2 together with the noisy gene expression model and parameter values as in [7] was used to generate 16,000 cells with different run speeds v_0, memory timescales t_M and run probabilities r_0. We varied D_R and D_T across cells to account for variations in the cell lengths and their effect on the rotational diffusion coefficients. Noting that the coefficient of variation (CV) of the *E. coli* cell lengths is $\sim 15\%$ [8], and that the rotational diffusion coefficient of an ellipsoid with major axis (length l) parallel to the direction of motion has $D_R \sim l^{-3}$ [9], we estimated the CV of D_R to be about 3 times that of the cell lengths, or $\sim 50\%$. Therefore, we sampled D_R from a log-normal distribution (to make sure $D_R > 0$) with mean $0.062\ s^{-1}$ [9] and standard deviation $0.03\ s^{-1}$. Assuming that D_T is affected similarly by the cell length, we fixed the ratio $D_T/D_R \approx 37$ across all cells. We simulated the cells in a quasi-linear gradient (fit from experimental data as described in [7]) of methyl-aspartate that varies from 0 to 1 mM over 10 mm. Near the bottom of the gradient (at $x = 1\ mm$) we calculated $L = 1500\ \mu m$ and near the top of the gradient (at $x = 9\ mm$) we found $L = 4800\ \mu m$.

In Fig 3 model 2 was used to generate 10^4 sample trajectories for each parameter set $\tau_{D0} = 1$ and $\tau_E = 0.1, 1, 3$, for a total time of 650 seconds in the exponential gradient, 2000 seconds in the linear gradient, and 3000 seconds for the localized source. We used constant run speed $v_0 = 20 \mu m/s$ and adapted probability to run $r_0 = 0.8$ for all cells and all parameter sets. In all cases the initial length scale where the cells start was $L_i = 1000\ \mu m$, from which we determined the cell memory t_M and the diffusion coefficients D_R and D_T using the timescale ratios.

References

1. Dufour YS, Fu X, Hernandez-Nunez L, Emonet T. Limits of feedback control in bacterial chemotaxis. PLoS Comput Biol. 2014 Jun;10:e1003694.

2. Si G, Wu T, Ouyang Q, Tu Y. Pathway-based mean-field model for *Escherichia coli* chemotaxis. Phys Rev Lett. 2012 Jul;109(4):048101.

3. Shimizu TS, Tu Y, Berg HW. A modular gradient-sensing network for chemotaxis in *Escherichia coli* revealed by responses to time-varying stimuli. Mol Syst Biol. 2010 Jun;6:382–395.

4. Sneddon MW, Pontius W, Emonet T. Stochastic coordination of multisple actuators reduce latency and improves chemotactic response in bacteria. Proc Natl Acad Sci U S A. 2012 Jan;109(2):805–810.

5. Tu Y. Quantitative modeling of bacterial chemotaxis signal amplification and accurate adaptation. Annu Rev Biophys. 2013 Feb;42:337–359.

6. Frankel NW, Pontius W, Dufour YS, Long J, Hernandez-Nunez L, *et al.* Adaptability of non-genetic diversity in bacterial chemotaxis. eLife. 2014 Oct;10.7554/eLife.03526.

7. Waite AJ, Frankel NW, Dufour YS, Johnston JF, Long J, *et al.* Non-genetic diversity modulates population performance. Mol Syst Biol. 2016 Forthcoming.

8. Taheri-Araghi S, Bradde S, Sauls JT, Hill NS, Levin PA, *et al.* Cell-Size Control and Homeostasis in Bacteria. Curr Biol. 2015 Feb;25:385–391.

9. Berg HC. Random walks in biology. Princeton: Princeton University Press; 1983.
