# Supplementary figures and images for: Feedback between motion and sensation provides nonlinear boost in run-and-tumble navigation
Source: PLoS Comput Biol. 2017 Mar 6;13(3):e1005429. doi: 10.1371/journal.pcbi.1005429 (PMC5358899; doi:10.1371/journal.pcbi.1005429)

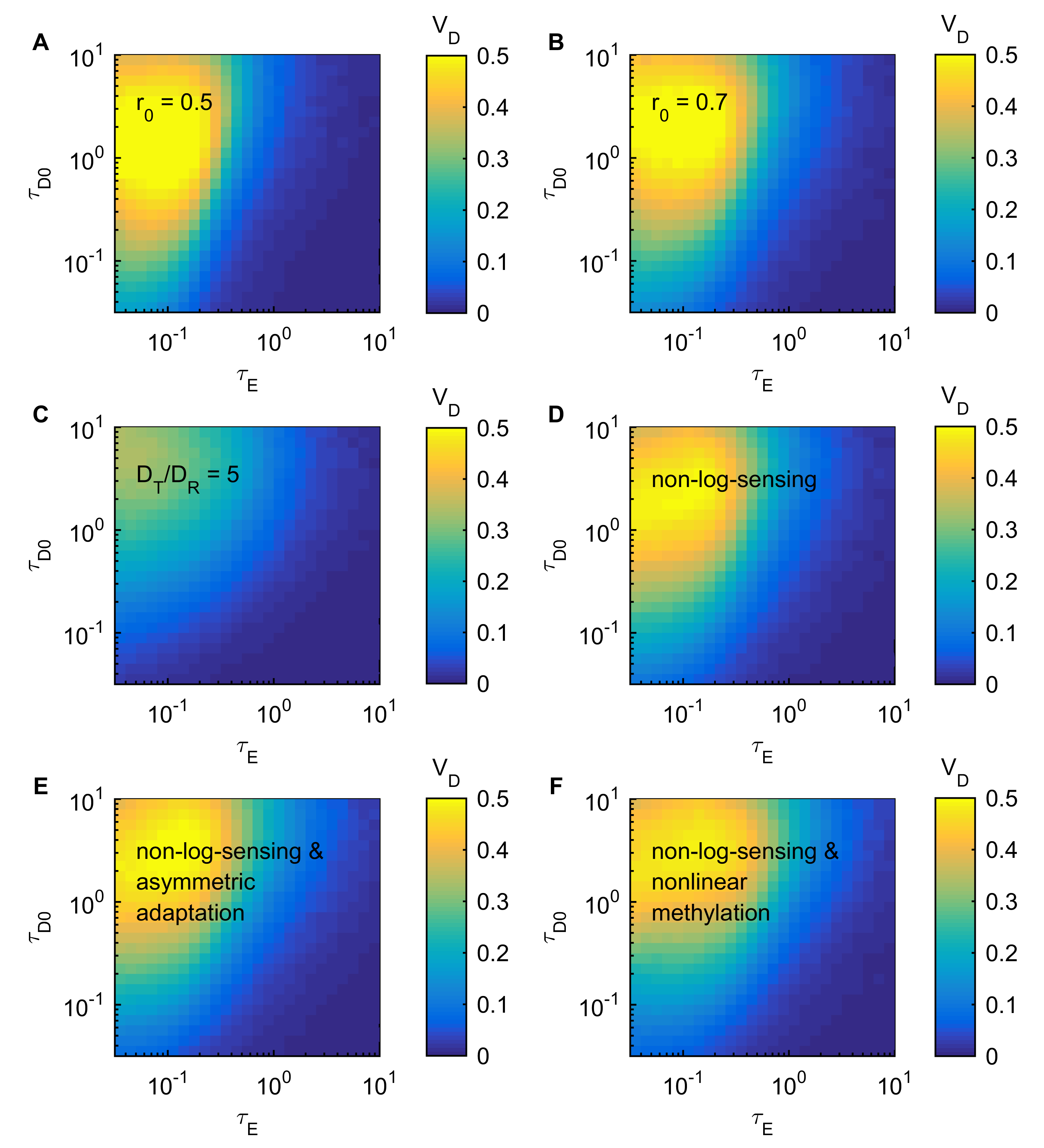

Supplement: S1 Fig — (A) Same as Fig 1A (where r0 = 0.8, Table S1 Table) except with r0 = 0.5. (B) r0 = 0.7. (C) Same as Fig 1A (where DT/DR ≈ 37, Table S1 Table) except with DT/DR = 5. (D) Same as Fig 1A except without assuming receptors in log-sensing range, i.e. Eq (S7) was used rather than Eq (S11). (E) Same as D, but additionally implements adaptation asymmetry, where the adaptation rate tM-1 in equation Eq (S4) depends on m [42]. Here the adaptation is 3 times faster when m > m(C) than when m < m(C), and tM is defined as the time scale when m < m(C). (F) Same as D but with nonlinear adaptation rate Eq (S4). Values for the parameters are: aB = 0.74, rB = 4.0, KR = 0.32, KB = 0.30, and VR and VB(0) chosen to ensure dmdt=0 when a = a0 and the adaptation time is tM when linearized. (TIF) [file pcbi.1005429.s002.tif]

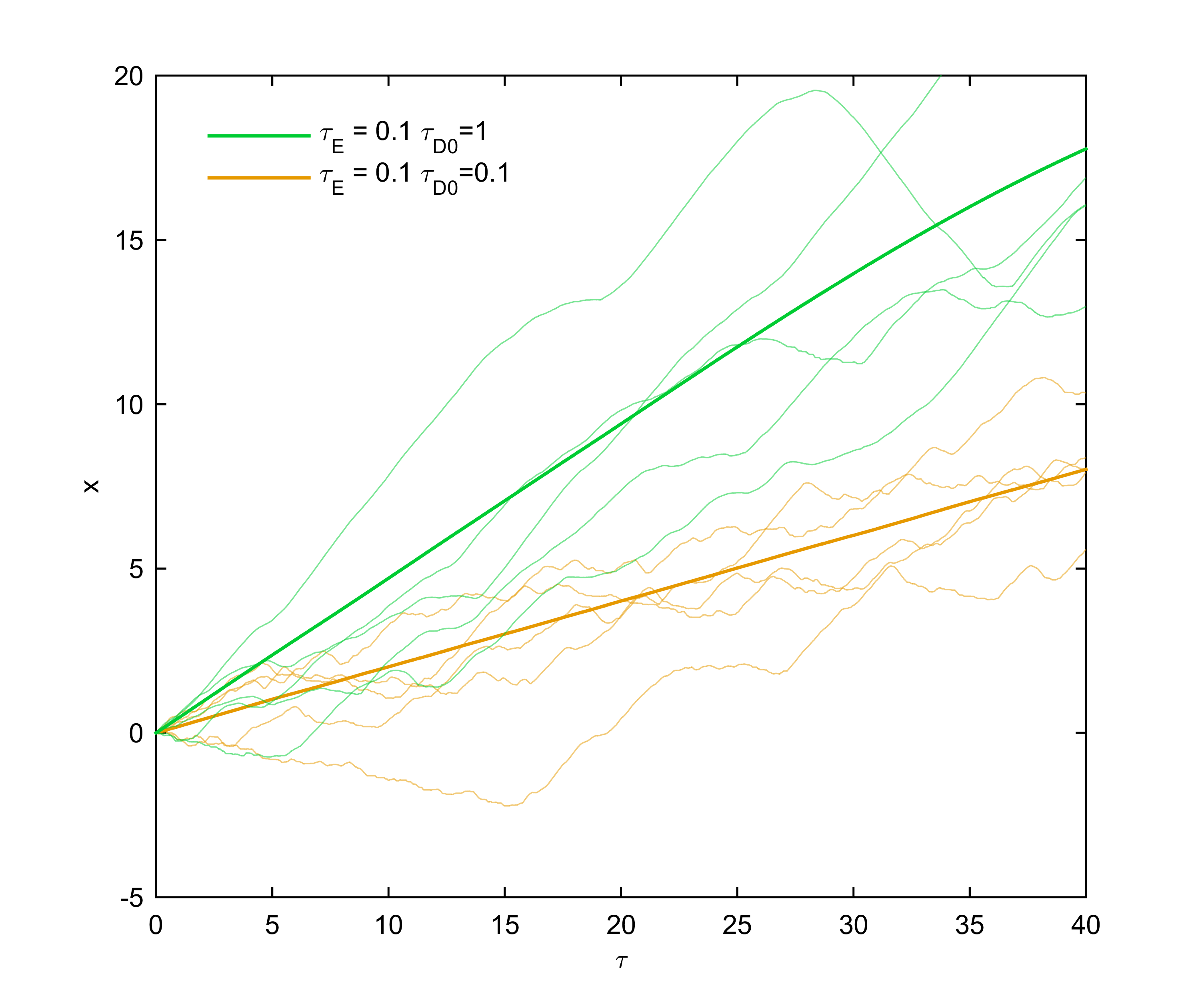

Supplement: S2 Fig — 5 sample trajectories (thin solid curves) and the mean over 104 sample trajectories (thick lines) of non-dimensionalized position x = X/(v0tM) as a function of time τ = t/tM. Colors correspond to cells with matching (green τD0 = 1) and non-matching (orange τD0 = 0.1) reorientation as in Fig 1 (same methods). (TIF) [file pcbi.1005429.s003.tif]

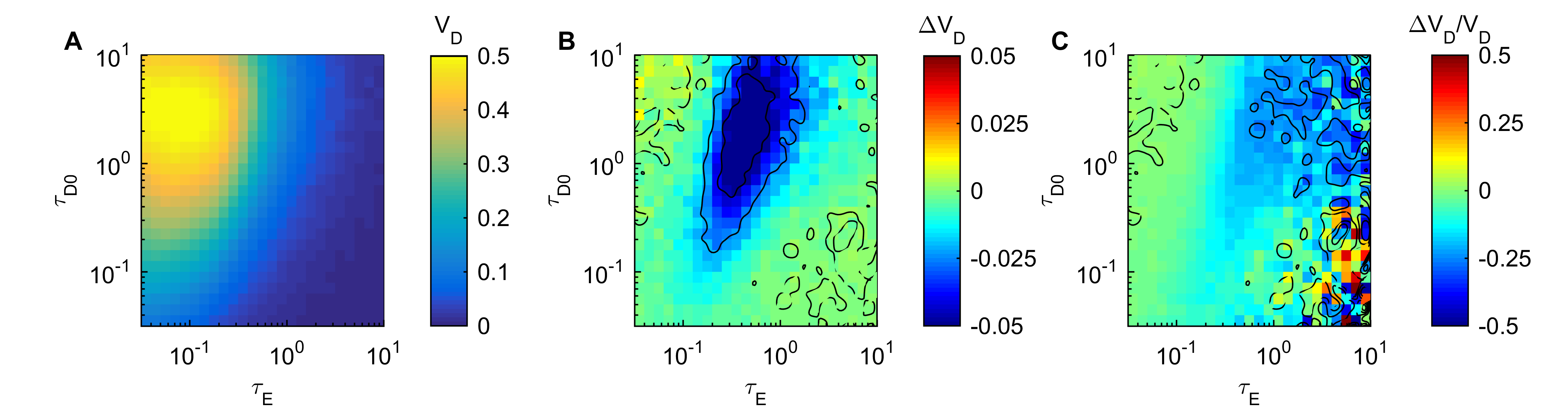

Supplement: S3 Fig — (A) Same as Fig 1A but adding a signaling noise term σm2/tMΓ(t) in Eq (S6) where Γ(t) is the standard Wiener process (see Eq [4] in [20]). From Eqs (S1)-(S2) and Y = αa we obtain dY/dm = Y(1 − a)ϵ1. Then plugging in σY/Y = 0.1 [20, 21], a ≈ 0.5 and ϵ1 = −1, we obtain σm = 0.2. (B) The absolute difference in drift speed between A (with signaling noise) and Fig 1A (without signaling noise), ΔVD = VD|noise − VD|nonoise, shows how signaling noise can either enhance or reduce the drift speed depending on (τE, τD). Note the colorbar is different. Black contour lines show level sets of ΔVD at the colorbar ticks (-0.05, -0.025, 0, 0.025, and 0.05). (C) The relative difference in drift speed (B divided by the drift speed without signaling noise). Again, the color scale is different and black contour lines show level sets at the colorbar ticks. (TIF) [file pcbi.1005429.s004.tif]
