# Supplementary material for: Feedback between motion and sensation provides nonlinear boost in run-and-tumble navigation
Source: PLoS Comput Biol. 2017 Mar 6;13(3):e1005429. doi: 10.1371/journal.pcbi.1005429 (PMC5358899; doi:10.1371/journal.pcbi.1005429)
Supplement: S1 Table — (PDF) [file pcbi.1005429.s005.pdf]

Table S1. Parameter values used in agent-based simulations.

**Fixed Parameters**			
Name	Definition	Value	References and Explanations
ϵ_0	Eq (S2)	6	Shimizu *et al.* 2010 [1]
ϵ_1	Eq (S2)	-1	Shimizu *et al.* 2010 [1]
N^{Rec}	Eq (S3)	6	Shimizu *et al.* 2010 [1]
K_i	Eq (S3)	0.0182 mM	Shimizu *et al.* 2010 [1]
K_a	Eq (S3)	3 mM	Shimizu *et al.* 2010 [1]
α	$Y = \alpha a$	6	Sneddon *et al.* 2012 [2]
K	Eq (S5)	3.06 mM	Sneddon *et al.* 2012 [2]
ϵ_2	Eq (S5)	40	Sneddon *et al.* 2012 [2]
ϵ_3	Eq (S5)	40	Sneddon *et al.* 2012 [2]
ω	Eq (S5)	1.3 s^{-1}	Sneddon *et al.* 2012 [2]
δ	Eq (S13)	-0.04	Equation Eq (S13)
H	Eq (S13)	4.9	Equation Eq (S13)
**Parameters in Fig 1A heatmap**			
Name	Definition	Value	References and Explanations
v_0	Maximal run speed	20 $\mu m/s$	Sneddon *et al.* 2012 [2]
r_0	$r(f = f_0)$	0.8	This study
t_M	Memory	10 s	Dufour *et al.* 2014 [3]
C_i	Initial concentraion	0.1 mM	This study
D_R	Rotational diffusion (run)	$0.00061 - 0.19$ s^{-1}	Let τ_{D0} vary from $10^{-1.5}$ to 10^1
D_T	Rotational diffusion (tumble)	$0.023 - 7.1$ s^{-1}	Keep $D_T/D_R \approx 37$ [4,5]
L	Gradient length scale	$186 - 58800$ μm	Let τ_E vary from $10^{-1.5}$ to 10^1
**Parameters in Fig 1A scatter plot that are different from [6]**			
Name	Definition	Value	References and Explanations
D_R	Rotational diffusion (run)	sampled from log-normal: mean 0.062 s^{-1} std 0.03 s^{-1}	Account for variations in cell lengths [4,7]
D_T	Rotational diffusion (tumble)	37 D_R	Keep $D_T/D_R \approx 37$ [4,5]
L	Gradient length scale	1500 μm and 4800 μm	$1/\|\|\nabla \ln C\|\|$
**Parameters in Fig 3**			
Name	Definition	Value	Reference and Explanations
v_0	Maximal run speed	20 $\mu m/s$	Sneddon *et al.* 2012 [2]
r_0	$r(f = f_0)$	0.8	This study
L_i	Initial gradient length scale	1000 μm	This study
C_0	Exponential gradient source	10 mM	This study
L_0	Length scale of C_0 source	1000 μm	This study
C_1	Linear gradient source	1 mM	This study
a_1	Slope of C_1 source	0.0001 $\mu M/\mu m$	This study
C_2	Localized source	1 mM	This study
R_0	Size of C_2 source	100 μm	This study
t_M	Memory	$0.54 - 17$ s	Let initial τ_E vary from 3 to 0.1
D_R	Rotational diffusion (run)	$0.0036 - 0.11$ s^{-1}	Keep $\tau_D = 1$ fixed
D_T	Rotational diffusion (tumble)	$0.13 - 4.2$ s^{-1}	Keep $D_T/D_R \approx 37$ [4,5]

References

1. Shimizu TS, Tu Y, Berg HW. A modular gradient-sensing network for chemotaxis in *Escherichia coli* revealed by responses to time-varying stimuli. Mol Syst Biol. 2010 Jun;6:382–395.

2. Sneddon MW, Pontius W, Emonet T. Stochastic coordination of multisple actuators reduce latency and improves chemotactic response in bacteria. Proc Natl Acad Sci U S A. 2012 Jan;109(2):805–810.

3. Dufour YS, Fu X, Hernandez-Nunez L, Emonet T. Limits of feedback control in bacterial chemotaxis. PLoS Comput Biol. 2014 Jun;10:e1003694.

4. Berg HC. Random walks in biology. Princeton: Princeton University Press; 1983.

5. Saragosti J, Silberzan P, Buguin A. Modeling *E. coli* tumbles by rotational diffusion. Implications for chemotaxis. PLoS ONE. 2012 Apr;7(4):e35412.

6. Waite AJ, Frankel NW, Dufour YS, Johnston JF, Long J, *et al.* Non-genetic diversity modulates population performance. Mol Syst Biol. 2016 Forthcoming.

7. Taheri-Araghi S, Bradde S, Sauls JT, Hill NS, Levin PA, *et al.* Cell-Size Control and Homeostasis in Bacteria. Curr Biol. 2015 Feb;25:385–391.
